# Estimating the population health burden of musculoskeletal conditions using primary care electronic health records

**DOI:** 10.1093/rheumatology/keab109

**Published:** 2021-02-09

**Authors:** Dahai Yu, George Peat, Kelvin P Jordan, James Bailey, Daniel Prieto-Alhambra, Danielle E Robinson, Victoria Y Strauss, Karen Walker-Bone, Alan Silman, Mamas Mamas, Steven Blackburn, Stephen Dent, Kate Dunn, Andrew Judge, Joanne Protheroe, Ross Wilkie

**Affiliations:** 1 Primary Care Centre Versus Arthritis, School of Medicine, Keele University; 2 MRC Versus Arthritis Centre for Musculoskeletal Health and Work, University of Southampton, Southampton; 3 Centre for Prognostic Research, Primary Care Centre Versus Arthritis, School of Primary, Community and Social Care, Keele University, Keele; 4 Centre for Statistics in Medicine, Nuffield Department of Orthopaedics, Rheumatology & Musculoskeletal Sciences, University of Oxford, Oxford; 5 MRC Lifecourse Epidemiology Unit, University of Southampton, Southampton; 6 Keele Cardiovascular Research Group, Centre for Prognosis Research, School of Medicine, Keele University, Keele; 7 Public Contributor; 8 Musculoskeletal Research Unit, University of Bristol, Bristol, UK

**Keywords:** electronic health records, primary care, musculoskeletal, health services research, surveillance, pain, quality of life, back pain, shoulder pain

## Abstract

**Objectives:**

Better indicators from affordable, sustainable data sources are needed to monitor population burden of musculoskeletal conditions. We propose five indicators of musculoskeletal health and assessed if routinely available primary care electronic health records (EHR) can estimate population levels in musculoskeletal consulters.

**Methods:**

We collected validated patient-reported measures of pain experience, function and health status through a local survey of adults (≥35 years) presenting to English general practices over 12 months for low back pain, shoulder pain, osteoarthritis and other regional musculoskeletal disorders. Using EHR data we derived and validated models for estimating population levels of five self-reported indicators: prevalence of high impact chronic pain, overall musculoskeletal health (based on Musculoskeletal Health Questionnaire), quality of life (based on EuroQoL health utility measure), and prevalence of moderate-to-severe low back pain and moderate-to-severe shoulder pain. We applied models to a national EHR database (Clinical Practice Research Datalink) to obtain national estimates of each indicator for three successive years.

**Results:**

The optimal models included recorded demographics, deprivation, consultation frequency, analgesic and antidepressant prescriptions, and multimorbidity. Applying models to national EHR, we estimated that 31.9% of adults (≥35 years) presenting with non-inflammatory musculoskeletal disorders in England in 2016/17 experienced high impact chronic pain. Estimated population health levels were worse in women, older aged and those in the most deprived neighbourhoods, and changed little over 3 years.

**Conclusion:**

National and subnational estimates for a range of subjective indicators of non-inflammatory musculoskeletal health conditions can be obtained using information from routine electronic health records.

**Rheumatology key messages**

- There is a lack of data to estimate the population burden of musculoskeletal conditions.
- We developed and validated models to estimate population musculoskeletal health using primary care electronic health records.
- The study adds new national and regional estimates of indicators of musculoskeletal health.

## Introduction

Musculoskeletal conditions such as low back pain (LBP) and osteoarthritis (OA) are extremely common, have proven over decades to be stubbornly resistant to treatment, and represent one of the greatest challenges to healthcare services and population health through their impact on everyday life [1]. Despite such overwhelming evidence of their significance, there is a lack of data that provide estimates of the extent of the impact of musculoskeletal conditions at a population level that can be used to guide interventions and preventative strategies.

Primary care electronic health records (EHR) offer the potential to be an ongoing source of data that can be used for surveillance and drive improvements in healthcare and health [2]. This ongoing collection of information can provide estimates of the number of people who have conditions and the processes of care such as the number that receive joint replacement or are prescribed pain medications and biologic therapies [2–5], although notably the availability of these data varies depending on geography and source (e.g. prescribed analgesics are well-recorded in primary care settings, joint replacement and biologic therapy are better recorded in secondary care data). However, the reason that people seek health care is not directly linked to the presence of musculoskeletal conditions but more so to the severity of symptoms (e.g. severity of pain) and their impact, in terms of disability and reduced quality of life [5], which drives the need for intervention and preventative strategies.

EHR does not routinely capture information on the severity or impact of musculoskeletal conditions and these data are best collected from patient reports [6, 7]. National surveys provide data on impact but have limited space for specific information on musculoskeletal conditions that can help with the prioritization of resources and services [2, 8, 9]. Combining EHR with patient reported information presents an opportunity to more accurately identify the impact of musculoskeletal conditions and the distribution and inequalities in the population [10]. However, patient reported information on musculoskeletal conditions may not always be available, and if EHR are to be used for ongoing surveillance, their ability to estimate the impact of musculoskeletal conditions must be examined [11].

In this study, the focus is on adults seeking healthcare for common musculoskeletal conditions. Five population indicators are proposed for surveillance of musculoskeletal health and that can be used to guide intervention strategies. The aim of this study was to examine if EHR data can estimate the extent of the impact of musculoskeletal conditions in musculoskeletal consulters at a population level.

## Methods

### Design

We conducted our investigation in three stages:

- A local census survey of all adults aged ≥35 years presenting to selected English general practices in one calendar year for non-inflammatory musculoskeletal conditions.
- Using linked primary care EHR data from consenting respondents, we derived and internally validated one model each for estimating population-level estimates of five self-reported indicators—the prevalence of high impact chronic pain, musculoskeletal health (mean Musculoskeletal Health Questionnaire (MSK-HQ) score), quality of life [mean EuroQoL health utility score (EQ-5D-5L)], prevalence of moderate-to-severe chronic LBP among LBP consulters, prevalence of moderate-to-severe chronic shoulder pain among shoulder pain consulters
- We applied our models using harmonized code lists to an independent national primary care EHR database (Clinical Practice Research Datalink) to obtain national and regional estimates of each indicator for three successive calendar years (2014/15, 2015/16 and 2016/17).

### Population and setting

The target population was adults aged ≥35 years presenting to primary care with LBP, neck pain, osteoarthritis, non-specific hip pain, knee pain, shoulder pain or hand/wrist pain.

### Musculoskeletal health indicators

Based on a review of national outcome frameworks [11, 12], existing indicators [13], proposed indicator sets for musculoskeletal health [14] and input from public contributors, we selected the following five musculoskeletal health indicators for this study:

- Proportion of MSK consulters with high impact chronic pain (HICP) defined as pain on most or all days in the previous 6 months and that limited life or work activities on most or all days. This approach is used in the US National Pain Survey [15].
- Mean Musculoskeletal Health Questionnaire (MSK-HQ) score: a 14-item questionnaire that captures key outcomes that patients with musculoskeletal conditions have prioritized as important for use across clinical pathways [16]. Scores range from 0 to 56, higher scores indicating better musculoskeletal health over the past 2 weeks [16].
- Mean EQ-5D-5L health utility score: the EQ-5D-5L self-classifier provides a self-reported description of health-related quality of life, rated on the day of response, according to a five-dimensional classification divided into five levels of perceived problem (no, slight, moderate, severe, unable). It has excellent psychometric properties [17]. We calculated the EQ-5D-5L utility score using the UK crosswalk value set [17], with scores ranging from <0.0 (representing health states worse than death) to 1.0 (full health).
- Proportion of LBP consulters with moderate-to-severe chronic LBP, defined as LBP present on most or all days in the previous 6 months *and* average intensity ≥5 on 0–10 NRS [18].
- Proportion of shoulder pain consulters with moderate-to-severe chronic shoulder pain, defined as shoulder pain present on most or all days in the previous 6 months *and* average intensity ≥5 on 0–10 NRS.

### Data sources

#### PRELIM survey-EHR linked dataset

As part of the PRELIM project (http://doi.org/10.21252/5ag3-ta31), we conducted a cross-sectional survey of all adults aged ≥35 years who had been registered for at least 10 years at one of 11 general practices in two Clinical Commissioning Groups (CCGs) in North Staffordshire and Stoke-on-Trent, UK, and who, between 1 July 2016 and 30 June 2017, had an eligible consultation for LBP, neck pain, osteoarthritis, non-specific hip pain, knee pain, shoulder pain, or hand/wrist pain using pre-defined Read (morbidity) code lists (available from www.keele.ac.uk/mrr). The total population of the 11 practices aged 35 years and over was 72 009 (26% of all 35+ year-olds served by the two CCGs). Forty per cent of the population of North Staffordshire live in rural areas while 99% of Stoke-on-Trent is urban. Thirty per cent of Stoke-on-Trent neighbourhoods are in the most deprived decile in England, but 10 neighbourhoods, mostly in North Staffordshire, are in the most affluent decile. Relative to England, the resident population has less ethnic diversity; 91% identify as White, with Asian/Asian British the next most common ethnic group comprising 9% of the population of Stoke-on-Trent.

We excluded patients with recorded inflammatory disease, spondyloarthropathy or crystal arthropathy. The survey instrument contained recommended items and instruments measuring the nature, severity and impact of MSK conditions, including the five indicators described above [17]. At 2 weeks, non-responders were re-sent the survey and offered the option of online completion, and at 4 weeks a minimum data collection survey was mailed to non-respondents, again with the option of online completion. Of 8461 mailed, 4528 responded (response rate 54%). Of these, 3828 (85%) consented to link their survey responses to routinely collected primary care EHR data, and 3710 (97%) had completed self-reported musculoskeletal health indicators. The general practices had all previously contributed to the CiPCA (North Staffordshire) primary care EHR database, which included training and assessment in morbidity recording [19], and been previously shown to give similar annual consultation prevalence rates for musculoskeletal conditions as national and international EHR databases [20, 21].

Covariates considered for inclusion in the models to estimate each of the five indicator measures were selected based on previous literature, expert opinion (including that of patients), potential association with MSK health status and routinely recorded within primary care EHR. These included demographic, socioeconomic, lifestyle, comorbidity, and musculoskeletal/pain-specific primary care contacts, diagnoses/problem codes, referrals, investigations and treatments (Table 1). A data manager independent from, and blinded to, survey data extracted these candidate covariates from the EHR of consenting respondents using pre-defined code lists (available from the authors; for the period up to 10 years prior to the survey). Details for definition of all candidate covariates are presented in Supplementary Table 1, available at *Rheumatology* online. Briefly, lifestyle predictors (i.e. smoking status, BMI), the most recent record before the index date was used; other candidate covariates were defined as having any record within 10 year prior to the survey (i.e. the Charlson Comorbidity Index was solely defined by Read codes, without combining other function or evaluation procedures).

**Table 1:** Descriptive characteristics and covariate distributions^a^ in musculoskeletal consulter cohorts, 2016–2017

	MSK consulters^b^ aged 35+ years, 2016–2017					Low back pain consulters aged 35+ years, 2016–2017					Shoulder pain consulters aged 35+ years, 2016–2017			
MSK health indicator(s) of interest	% with high impact chronic pain, mean MSK-HQ score, mean EQ-5D-5L score					% with moderate-to-severe chronic low back pain					% with moderate-to-severe chronic shoulder pain			
Data source	PRELIM Survey-EHR		CPRD			PRELIM Survey-EHR		CPRD			PRELIM Survey-EHR		CPRD	
*n*	3710		49 788			1046		15 153			604		9690	
Age, median (IQR), years	67 (57–75)		61 (52–72)			65 (55–74)		59 (49–71)			66 (57–74)		61 (52–72)	
Female	2191 (59)		28 880 (58)			620 (59)		8907 (59)			346 (57)		5564 (57)	
Index of multiple deprivation														
Quintile 1 (most deprived)	626 (17)		6909 (14)			185 (18)		2524 (17)			110 (18)		1314 (14)	
Quintile 2	575 (15)		7818 (16)			157 (15)		2630 (17)			83 (14)		1469 (15)	
Quintile 3	748 (20)		8988 (18)			211 (20)		2813 (19)			120 (20)		1745 (18)	
Quintile 4	1184 (32)		10 153 (20)			328 (31)		2949 (19)			196 (33)		1993 (21)	
Quintile 5 (least deprived)	630 (17)		15 920 (32)			165 (16)		4237 (28)			95 (16)		3169 (33)	
MSK site-specific pain/condition														
Neck pain	897 (24)		11 487 (23)			211 (20)		2465 (16)			120 (20)		1798 (19)	
Shoulder pain	1129 (30)		16 306 (33)			221 (21)		3044 (20)			—		—	
Back pain	1793 (48)		25 559 (51)			—		—			199 (33)		3603 (37)	
Hip pain	847 (23)		7583 (15)			199 (19)		2054 (14)			87 (14)		1242 (13)	
Knee pain	1574 (42)		21 876 (44)			293 (28)		3668 (24)			192 (32)		2597 (27)	
Hand pain	1008 (27)		9483 (19)			205 (20)		1724 (11)			125 (21)		1258 (13)	
Osteoarthritis	1445 (39)		9894 (20)			259 (25)		1634 (11)			189 (31)		1337 (14)	
Time since 1st MSK consultation, median (IQR), days: median (IQR)	1179 (483–1590)		1053 (310–1549)			1228 (518–1620)		1,094 (323–1563)			1,283 (552–1,629)		1,066 (316–1,562)	
Time since last MSK consultation, median (IQR), days: median (IQR)	132 (97–162)		150 (66–253)			131 (97–161)		143 (59–245)			136 (97–162)		140 (59–240)	
Number of MSK consultations: , median (IQR)	5 (2–9)		3 (2–6)			5 (3–10)		3 (2–6)			5 (3–10)		4 (2–6)	
Any analgesic prescription	3,051(82)		36 048(72)			877(84)		11 983(79)			485(80)		6,828(71)	
Highest-level of analgesic prescription:														
No analge*sic*sics	659(18)		13 740(28)			169(16)		3,170(21)			119(20)		2,862(30)	
Ba*sic*sic analge*sic*sics	561(15)		5,445(11)			113(11)		1,101(7)			82(14)		1,055(11)	
Weak analge*sic*sics	420(11)		4,469(9)			115(11)		1,326(9)			69(11)		808(8)	
Moderate analge*sic*sics	424(11)		3,834(8)			127(12)		1,264(8)			67(11)		743(8)	
Strong/very strong analge*sic*sics	1,646(44)		22 300(45)			522(50)		8,292(55)			267(44)		4,222(44)	
NSAIDsS prescription	1,331(35)		25 878(52)			428(41)		8,637(57)			223(37)		5,270(54)	
Antidepressant prescription	1,396(37)		28 615(58)			465(45)		9,319(62)			240(40)		5,578(58)	
Sedative prescription	221(6)		6,446(13)			73(7)		2,158(14)			41(7)		1,322(14)	
Muscle relaxant prescription	51(1)		8,582(17)			24(2)		3,717(25)			5(1)		1,530(16)	
Any MSK referral	1,400(37)		31 416(63)			404(39)		9,169(61)			237(39)		6,516(67)	
MSK X-ray	2,107(56)		19 160(38)			492(47)		5,919(39)			339(56)		1,468(15)	
MSK MRI	121 (3)		2745 (6)			53 (5)		1293 (9)			19 (3)		215 (2)	
MSK surgery	527 (14)		8179 (16)			137 (13)		1954 (13)			68 (11)		1943 (20)	
Joint injection	308 (8)		3847 (8)			75 (7)		1027 (7)			76 (13)		1144 (12)	
Fracture	157 (4)		3348 (7)			49 (5)		1006 (7)			20 (3)		622 (6)	
Impact on work (recorded fit-note)	956 (25)		4067 (8)			326 (31)		1408 (9)			152 (25)		782 (8)	
BMI														
Healthy/underweight (<25 kg/m^2^)	320 (9)		11 022 (22)			109 (10)		3494 (23)			69 (11)		2191 (23)	
BMI not recorded	535 (14)		10 880 (22)			150 (15)		3325 (22)			69 (11)		2064 (21)	
Overweight (25–29.9 kg/m^2^)	1603 (43)		13 871 (28)			642 (61)		4163 (28)			395 (65)		2774 (29)	
Obese (≥30 kg/m^2^)	1252 (34)		14 074 (28)			124 (12)		4171 (28)			71 (12)		2661 (28)	
Smoking status														
Non-smoker	1435 (39)		21 390 (43)			351 (34)		6120 (40)			244 (40)		4270 (44)	
Smoking status not recorded	324 (9)		5726 (12)			254 (24)		1669 (11)			46 (8)		1066 (11)	
Ex-smoker	716 (19)		14 257 (29)			156 (15)		4328 (29)			114 (19)		2765 (29)	
Current smoker	1235 (32)		8415 (17)			285 (27)		3036 (20)			200 (33)		1589 (16)	
Drinking status														
Non-drinker/drinking status not recorded/ex-drinker	1785 (48)		2470(53)			213 (20)		8237 (54)			116 (19)		5120 (53)	
Current Drinker	1925 (52)		23 408 (47)			833 (80)		6916 (46)			488 (81)		4570 (47)	
Charlson Comorbidity Index (0-–37)	0 (0–2)		0 (0–1)			0 (0–1)		0 (0–1)			0 (0–1)		0 (0–1)	
eFI score (0-–10): ), median (IQR)	0.08 (0.06–0.14)		0.06 (0.03–0.11)			0.08 (0.06–0.14)		0.06 (0.03–0.11)			0.08 (0.03–0.14)		0.06 (0.03–0.08)	
Anxiety or depression consultation	751(20)		10 736(22)			253(24)		3,759(25)			118(20)		2,123(22)	

Data are *n* (%), unless otherwise stated. Mean MSK-HQ and Mean EQ-5D-5L are outcomes (indicators) were both derived from MSK consulter cohorts. ^a^Covariates were defined within a 5-year look back period in each cohort members’ electronic health record (i.e. 60 months prior to 30 June 2017). ^b^Defined as non-specific, non-inflammatory low back pain, neck pain, shoulder pain, hand/wrist pain, hip pain, knee pain or osteoarthritis. eFI: electronic frailty index; IQR: interquartile range; MSK: musculoskeletal.

**Table 2:** England national and subnational estimates for MSK health indicators based on application of final models

	MSK consulters^a^ aged 35+ years									Low back pain consulters aged 35+ years			Shoulder pain consulters aged 35+ years		
MSK health indicator	% with high impact chronic pain			Mean MSK-HQ score (0–56)			Mean EQ-5D-5L score (−0.224 to 1)			% with moderate- to-severe chronic back pain			% with moderate- to-severe chronic shoulder pain		
Year	2014/15	2015/16	2016/17	2014/15	2015/16	2016/17	2014/15	2015/16	2016/17	2014/15	2015/16	2016/17	2014/15	2015/16	2016/17
Overall (crude)	33.3	32.1	31.9	33.5 (7.8)	33.7 (7.9)	33.8 (8.0)	0.60 (0.15)	0.62 (0.15)	0.66 (0.14)	27.4	25.8	26.0	29.7	27.4	27.8
Men	31.6	30.6	30.4	34.5 (7.9)	34.6 (8.0)	34.8 (8.0)	0.62 (0.14)	0.63 (0.15)	0.67 (0.14)	25.9	24.0	24.4	28.1	25.8	26.6
Women	34.6	33.2	33.0	32.8 (7.7)	33.0 (7.8)	33.2 (7.9)	0.59 (0.15)	0.61 (0.15)	0.66 (0.15)	28.5	27.0	27.2	31.0	28.6	28.7
Age Group															
35–44 years	30.2	30.0	29.2	32.9 (8.0)	32.8 (8.1)	33.1 (8.2)	0.64 (0.15)	0.64 (0.15)	0.73 (0.13)	27.4	26.5	25.7	25.5	23.9	23.8
45–54 years	29.4	28.9	28.9	34.0 (8.2)	34.2 (8.2)	34.1 (8.4)	0.65 (0.15)	0.65 (0.15)	0.72 (0.14)	25.9	24.6	25.5	26.2	24.2	24.1
55–64 years	30.2	28.8	28.9	34.6 (8.1)	34.8 (8.2)	34.9 (8.3)	0.64 (0.14)	0.65 (0.15)	0.71 (0.14)	26.4	24.6	25.1	28.7	26.5	27.4
65–74 years	33.5	32.2	32.2	33.9 (7.7)	34.1 (7.7)	34.2 (7.8)	0.61 (0.14)	0.62 (0.14)	0.68 (0.14)	26.9	25.1	25.6	31.6	29.3	30.2
75–84 years	39.7	37.8	37.8	32.1 (7.0)	32.3 (7.0)	32.5 (7.2)	0.57 (0.14)	0.57 (0.14)	0.64 (0.14)	30.0	27.9	27.3	34.8	31.1	31.7
85+ years	45.5	44.1	44.5	30.4 (6.2)	30.3 (6.3)	30.3 (6.2)	0.50 (0.14)	0.51 (0.13)	0.58 (0.14)	33.4	31.3	32.2	31.7	31.0	30.1
Index of multiple deprivation															
Quintile 1 (most)	42.9	42.2	41.4	28.4 (6.4)	28.3 (6.5)	28.6 (6.6)	0.54 (0.15)	0.52 (0.14)	0.62 (0.15)	37.5	35.7	36.3	33.3	30.7	30.5
Quintile 2	33.3	32.5	32.0	32.3 (7.3)	32.3 (7.3)	32.4 (7.3)	0.59 (0.14)	0.60 (0.14)	0.68 (0.14)	23.3	22.1	21.7	29.7	29.0	29.6
Quintile 3	36.4	35.2	35.1	33.2 (7.4)	33.4 (7.4)	33.3 (7.5)	0.60 (0.14)	0.61 (0.14)	0.68 (0.14)	27.7	26.9	26.7	30.1	27.9	28.5
Quintile 4	31.2	29.9	30.2	34.6 (7.6)	34.7 (7.6)	34.7 (7.6)	0.64 (0.14)	0.64 (0.14)	0.70 (0.14)	25.9	23.9	24.4	28.8	26.0	27.7
Quintile 5 (least)	27.4	26.6	27.1	36.5 (7.8)	36.4 (7.9)	36.4 (7.9)	0.69 (0.14)	0.67 (0.14)	0.72 (0.13)	24.8	23.2	23.3	28.1	25.7	25.4
Region															
North East	37.6	35.5	39.8	31.7 (7.5)	31.9 (7.1)	30.6 (7.4)	0.58 (0.15)	0.59 (0.14)	0.65 (0.16)	31.6	30.3	38.5	31.1	31.2	39.3
North West	36.2	34.8	34.0	31.9 (7.5)	32.1 (7.6)	32.4 (7.7)	0.59 (0.14)	0.59 (0.15)	0.68 (0.14)	30.0	28.2	26.6	31.3	27.8	27.3
Yorkshire and Humber	36.8	32.8	30.9	31.8 (7.4)	32.8 (7.7)	33.4 (7.7)	0.59 (0.14)	0.61 (0.14)	0.71 (0.14)	28.3	23.2	23.9	32.3	29.2	26.2
East Midlands	—	—	—	—	—	—	—	—	—	—	—	—	—	—	—
West Midlands	33.5	32.8	33.2	33.5 (7.7)	33.2 (7.8)	33.2 (7.7)	0.62 (0.15)	0.64 (0.15)	0.68 (0.14)	28.3	27.9	28.5	30.9	28.5	29.0
East of England	31.3	30.0	30.5	34.5 (7.8)	34.9 (8.0)	34.5 (8.2)	0.63 (0.15)	0.61 (0.15)	0.70 (0.14)	27.1	24.9	24.3	30.4	28.1	28.6
South West	35.7	34.4	35.2	32.7 (7.8)	32.9 (7.8)	32.5 (7.9)	0.60 (0.15)	0.61 (0.15)	0.68 (0.15)	29.6	28.6	29.5	31.1	29.4	31.1
South Central	31.6	30.5	29.4	34.5 (7.9)	34.7 (7.9)	35.3 (8.0)	0.63 (0.14)	0.64 (0.15)	0.70 (0.14)	25.9	24.0	25.1	29.2	26.4	28.7
London	31.8	30.8	30.8	33.8 (8.0)	33.8 (8.0)	33.9 (8.1)	0.62 (0.14)	0.62 (0.15)	0.69 (0.15)	25.9	24.4	24.3	27.9	26.1	24.8
South East Coast	32.0	31.4	31.1	34.2 (8.0)	34.1 (7.9)	34.4 (8.0)	0.63 (0.14)	0.63 (0.15)	0.70 (0.14)	25.1	24.3	24.7	27.3	26.6	26.6

Data source: Clinical Practice Research Datalink (CPRD). Covariates were defined using 5-year look back period. ^a^Defined as non-specific, non-inflammatory low back pain, neck pain, shoulder pain, hand/wrist pain, hip pain, knee pain, or osteoarthritis. EQ-5D-5L: EuroQoL 5 dimensions, 5-level version; MSK: musculoskeletal; MSK-HQ: Musculoskeletal Health Questionnaire.

These data were then linked to survey data to create the PRELIM Survey.

#### Clinical practice research datalink national EHR data

Clinical practice research datalink (CPRD) GOLD contains EHR data from over 10 million patients registered with over 650 UK general practices [22]. For this study we used data from practices (all in England) which consented to linkage to the Index of Multiple Deprivation (IMD) [23]. Based on patient’s residential postcode, IMD is a composite measure of neighbourhood deprivation incorporating domains on income, employment, education, health, housing, crime, and environment. Using code lists for eligibility criteria that were harmonized with those used in PRELIM Survey-EHR we included adults aged ≥35 years (*n* = 49 788) consulting for a non-inflammatory musculoskeletal pain condition in July 2016–June 2017 (i.e. as per PRELIM). Using another set of harmonized code lists we extracted information on their covariates recorded in the previous 10 years. We then repeated this process for cases consulting between July 2015 and June 2016 and between June 2014 and July 2015 to evaluate the stability over time of our modelled estimates.

### Statistical analysis

#### Model development and internal validation

Using data from the PRELIM Survey-EHR data, we derived and internally validated multivariable models for each indicator. Multivariable logistic regression was used to model the three binary indicators (high impact chronic pain, moderate-to-severe chronic LBP, moderate-to-severe chronic shoulder pain). Multivariable linear regression was used to model MSK-HQ and EQ-5D-5L scores with these two indicators first transformed (MSK-HQ^0.5^, e^EQ-5D-5L^). For the comorbidity and prescription covariates, the lack of a record was presumed as absence (i.e. no diagnosis or prescription). For BMI, missing data were categorized as ‘not recorded’, along with the categories of healthy/underweight (BMI < 25 kg/m^2^), overweight (25 kg/m^2^≤BMI < 30 kg/m^2^), and obese (BMI ≥ 30 kg/m^2^). Similarly, ‘not recorded’ was added as a category for drinking and smoking status. Multiple imputation was not used as the absence of a record of these covariates may be associated with the value of the outcome indicator. Multivariable fractional polynomials were used for modelling potential non-linear relationships between continuous covariates and outcomes.

We first determined the optimal number of years prior to MSK consultation (the ‘look-back’ period) needed to identify covariates in the EHR. For each indicator we fitted 10 full models (all covariates included) using 1–10 years of retrospective EHR data to define the covariates. The look-back period with best model performance after assessment of Akaike information criterion, Bayesian information criterion, *R*^2^ and C-statistics (for binary indicators) was chosen. For the final parsimonious models using the optimal look-back period, covariates were dropped through backward stepwise elimination (*P* *>* 0.2, based on change in log likelihood), with age and gender retained in all models. Finally, interactions of included covariates with age were assessed to see if they improved the model.

#### Model performance

For subgroups of the population based on age, gender, CCG and deprivation, we compared the observed prevalence rates and mean scores (as appropriate) of the indicators from the PRELIM survey with their estimated values derived from the models utilizing the linked EHR. For logistic regression models, performance of the final model was also examined using the C-statistic. For linear regression models, performance was assessed using *R*^2^ (proportion of the variance in continuous outcomes explained by the included covariates).

Final models were applied to 100 bootstrapped samples to examine performance (as described above), and then to the original dataset to test model performance and optimism (the difference in the performance in the bootstrapped and original data). Overall optimism was estimated for all models. The overall optimism-corrected calibration of these models was assessed graphically by plotting agreement between predicted and observed values for each decile of predicted risk.

#### Application of models to national EHR data

The final parsimonious, optimism-corrected models derived in the PRELIM Survey-EHR data were then applied to the relevant MSK consulter cohorts in the CPRD dataset to estimate the prevalence/mean of each of the five indicators for national estimates in three consecutive years: 2014/15, 2015/16 and 2016/17. For the three binary indicators, the estimated prevalence was the mean of the estimated individual probabilities in the specific population. For the continuous indicators, the estimated mean was the mean of the estimated individual scores [transformed back from estimates in the linear regression, as (MSK-HQ^0.5^)^2^ for MSK-HQ and ln(e^EQ-5D-5L^)] for EQ-5D-5L in the specific population. We present these estimates overall, and stratified by sex, age (10-year age bands), deprivation (quintiles) and geographical region.

To explore the sensitivity of our findings to length of look-back period, we repeated all the preceding steps using a 2-year look-back period in the EHR data.

### Ethical approval

Ethical approval was obtained for the PRELIM survey and linkage to primary care EHR data from the North West-Greater Manchester East Research Ethics Committee (REC Ref: 15/NW/0735). The use of CPRD was approved by the Independent Scientific Advisory Committee (reference number: 18_014).

### Patient and public involvement

Public contributors were involved throughout this study to ensure that the perspectives of patients remained at the centre of the research. Ten public contributors from the Research User Group, Keele University, were involved in the study, as part of advisory groups or study management meetings. They provided patient perspectives on the development of the proposal (particularly on linkage of data from EHR and questionnaires), study materials (participation information sheets, consent forms) and the PRELIM questionnaire. A public co-applicant (S.D.) is a member of the study team and two other public contributors attended the study steering committee.

## Results

### Model development and apparent performance—PRELIM Survey-EHR

Based on consistently good relative model fit and performance, the 5-year look-back period for identifying covariates recorded in the EHR was selected as optimal for all indicators, although differences between look-back periods were generally small (Supplementary Fig. 1, available at *Rheumatology* online). Distribution of the covariates over the 5-year period in the PRELIM Survey-EHR cohort are given in Table 1.

After backward elimination, between 7 and 16 covariates were retained in each model (minimum of 14 events per parameter in logistic regression models and 143 subjects per parameter in linear regression models). The coefficients of the models are given in Supplementary Table 2, available at *Rheumatology* online. Prescription of strong or very strong analgesia was strongly associated with all five indicators while antidepressant prescriptions, time since MSK consultation and area-level deprivation were strongly associated with four of the five indicators. Any MSK referral and joint injection were associated with moderate-to-severe chronic low back pain and EQ-5D-5L, respectively. MSK X-ray and smoking were associated with moderate-to-severe chronic shoulder pain. The non-linear associations of continuous covariates with indicators is shown in Supplementary Fig. 2, available at *Rheumatology* online.

**Fig. 1:**
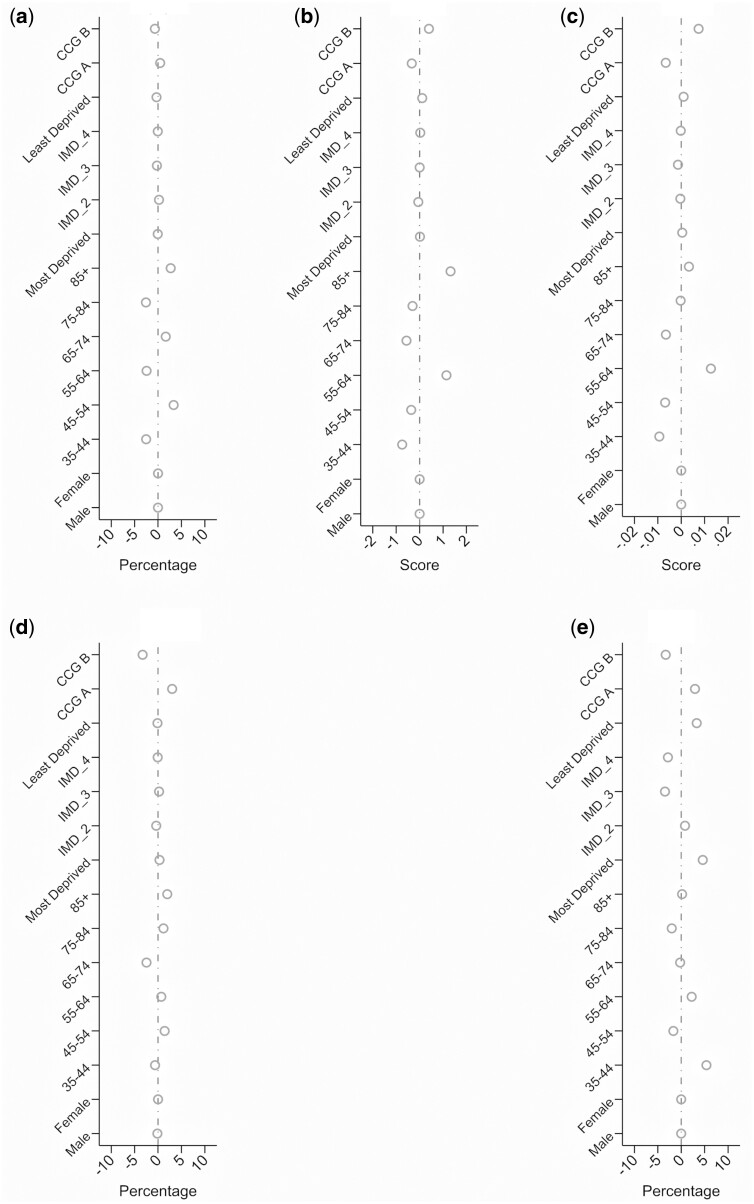
Difference between observed and estimated prevalence/mean score for each MSK Health indicator by gender, age, deprivation and Clinical Commissioning Group (CCG) (**A**) High impact chronic pain. (**B**) Moderate-to-severe chronic low back pain. (**C**) Moderate-to-severe chronic shoulder pain. (**D**) MSK-HQ score. (**E**) EQ-5D-5L score.

Absolute differences between observed and estimated prevalence rates and means when stratified by age, sex, CCG and deprivation are presented in Fig. 1. Estimated prevalence varied from that observed by a maximum of 5% for high impact chronic pain, moderate-to-severe chronic shoulder pain and moderate-to-severe chronic LBP; and mean scores by ±0.2 for MSK-HQ score and ±0.01 for EQ-5D-5L score. The optimism-corrected C-statistics for the three prediction models for binary MSK health indicators ranged from 0.74 to 0.77, while for the two continuous indicators the optimism-corrected *R*^2^ values were 0.30 and 0.33 (Supplementary Table 3, available at *Rheumatology* online). The optimism-corrected calibration slopes were all 0.99 and with good agreement between observed and estimated prevalence rates and means.

### National estimates of MSK indicators

Compared with MSK consulters recorded in CPRD, participants in the PRELIM Survey-EHR cohort were older, and more likely to live in deprived neighbourhoods (Table 1). They were also more likely to have previous recorded MSK consultations in the hand and hip and for osteoarthritis, analgesic prescriptions and MSK X-ray. However, the level of recorded prescriptions for NSAIDs, antidepressants, muscle relaxants and sedatives as well as MSK referrals were lower.

By applying our final PRELIM-derived models in CPRD, we estimated nationally that 31.9% of adults aged 35 years and over who had consulted for a common non-inflammatory musculoskeletal pain condition in 2016–2017 would be experiencing high impact chronic pain (Table 2). The estimated mean MSK-HQ and EQ-5D-5L scores in these MSK consulters were 33.8 and 0.66, respectively. Among recent LBP consulters, an estimated 26.0% had moderate-to-severe chronic LBP. Of recent shoulder pain consulters, an estimated 27.8% had moderate-to-severe chronic shoulder pain. Across all indicators, MSK health among consulters was worse in women than in men, with older age, and in those living in the most deprived neighbourhoods. Over the three consecutive years from 2014/15 to 2016/17 age-, sex- and deprivation-specific estimates for all indicators showed either no or small improvements with greatest increases seen in mean EQ-5D-5L scores in all strata. The sensitivity analysis using a shorter 2-year look-back period for covariates gave similar estimates and patterns, although a slightly lower prevalence of high impact chronic pain (28.9% *vs* 31.9% in 2016/17) and a slightly higher prevalence of moderate-to-severe chronic LBP (29.2% *vs* 26.0%) (Supplementary Table 4, available at *Rheumatology* online).

## Discussion

### Summary of main findings

Our study provides evidence that it is feasible to use routinely collected EHR data to estimate the extent of the impact of musculoskeletal conditions in populations to guide interventions and healthcare planning. While the remit of our study was specifically five selected indicators on the severity and impact of common, non-inflammatory musculoskeletal disorders, the methodology is likely to be generalizable to other indicators and other musculoskeletal conditions.

### Comparison with previous research

To our knowledge this is the first study to use prediction model methodology based on routine EHR data to estimate the prevalence and distribution of patient-reported severity and impacts of musculoskeletal conditions. Efforts to classify the severity of long-term musculoskeletal conditions from information in the EHR [24] are based on the expectation that severity can be meaningfully inferred from available patterns of coded events and processes. Our approach extends this by directly modelling patient-reported measurement of severity to obtain population-level estimates of health. Primary care EHRs currently contain little systematic measurement of pain severity, functional status, wellbeing and quality of life. As a result, there are few direct comparisons for the estimates provided here. UK and US surveys estimate the prevalence of moderately severely disabling chronic pain/high impact chronic pain in the adult general population at between 10 and 16% [25]. Our estimate of 32% with high impact chronic pain among MSK consulters aged over 35 years reflects the older age range in our study but more crucially the selection of a high-risk group (MSK consulters). Where comparable estimates exist in MSK consulter populations, our estimates appear similar. For example, our estimated mean MSK-HQ and EQ-5D-5L scores of 33.8 and 0.66 among MSK consulters were just slightly higher (indicating better MSK health) than those reported in a study of adult musculoskeletal patients referred to community physiotherapy clinics (30.5 and 0.60, respectively) [26]. Our estimated EQ-5D-5L mean score is higher than that from the General Practice Patient Survey (0.577) [13], which is likely to reflect the fact that the former is restricted to adults reporting a long-term MSK problem. The current indicator for the prevalence of ‘severe back pain’ used in the PHE Fingertips tool is also applied to those with a long-term back problem and uses a lower threshold for defining ‘severe’. Our estimates show the expected pattern of worse MSK health in females, older ages, and those living in more deprived neighbourhoods.

### Strengths and limitations

Our study illustrates an approach to producing timely, affordable indicators of the non-fatal impacts of musculoskeletal conditions that could be derived from continuous EHR data at national and subnational levels. It highlights the potential benefits of such an approach to inform health system responses to the growing challenge of musculoskeletal conditions, which have historically received less attention than other conditions. We deliberately focused on the subpopulation of adults aged ≥35 years who had a record of a non-inflammatory MSK consultation in the previous year. Our estimates do not therefore cover younger ages or those suffering MSK conditions but not presenting to primary healthcare in a given year of interest. Our survey, designed with the involvement of patients and members of the public, provided rich self-reported information on musculoskeletal health from nearly 4000 adults, with a response rate equivalent to that of the Health Survey for England (HSE) [27], and substantially higher than the national GP Patient Survey [28]—both sources currently used to produce national musculoskeletal health indicators in England. A high proportion of respondents consented to EHR linkage in practices with a history of high-quality coding. Our public contributors improved the clarity of the study materials for participants and identified key areas for inclusion in the study questionnaire. Our public co-applicant (and co-author) provided the patient perspective on study decision-making. However, our local sampling frame is known to under-represent black, Asian and minority ethnic groups compared with the national average. Future enriched sampling of these groups or a shift to nationally representative survey sample frames with EHR linkage is needed. We found that models based on 5 years of continuous retrospective records were generally optimal but excluding patients and practices with <5 years’ prior registration does reduce the sample size and has the potential to introduce selection bias. We used 5 years for all models for consistency. Other indicators or conditions may require fewer years of continuous records. In our study, models requiring only 2 years of retrospective records were only marginally inferior and we have provided these in full in Supplementary data, available at *Rheumatology* online. The models rely on consistent coding of the included covariates. Lifestyle information, in particular, can often be missing from these records, but completeness has been improving over recent years. Performance of models could be improved by including information from the unstructured free text within the EHR [29] but access to this is increasingly difficult for researchers in the UK due to information governance restrictions. The prediction models have been derived using retrospective data and are limited in their application at the individual level to identify those at high risk. A prospective cohort design would be able to yield more discriminated and calibrated prediction model to identify high-risk individuals.

### Implications for research and practice

The need to integrate patient-reported outcomes into EHRs has received considerable attention, but typically from the standpoint of clinical care and organisation of health services. We hope that our study stimulates further research on the harnessing of data within the EHR for population musculoskeletal health indicators and greater attention within health policy and practice to preventing and reducing disability associated with common musculoskeletal conditions in the population. Our national estimates confirm the significant impact of musculoskeletal pain. Future external validation of our models, including research that explores how frequently such models may need to be updated in response to changing patterns of healthcare use and recording, and validation in other geographical areas with health record collation and linkage (such as in Scotland and Wales), are encouraged. Future studies using EHR to estimate the impact of MSK conditions on work ability are also warranted.

## Conclusion

Information routinely recorded within English care EHR can estimate the prevalence and extent of key patient-reported measures of musculoskeletal health among adult consulters with acceptable accuracy. This approach could provide a sustainable, timely source for a richer array of population musculoskeletal health indicators to inform and support health policy and practice.

## Supplementary Material

keab109_supplementary_data
