# Supplementary material for: Estimating the population health burden of musculoskeletal conditions using primary care electronic health records
Source: Rheumatology (Oxford). 2021 Feb 9;60(10):4832–43. doi: 10.1093/rheumatology/keab109 (PMC8487274; doi:10.1093/rheumatology/keab109)
Supplement: keab109_supplementary_data [file keab109_supplementary_data.zip › rhe-20-2578-File003.docx]

**Supplemental Table-1.** Description of all candidate predictors used in models

| **NO** | **PREDICTORS** | **DESCRIPTION** |
| --- | --- | --- |
| 1 | Age | Continuous variable |
| 2 | Gender | Binary variable (Female VS. Male) |
| 3 | MSK contacts-1 | Continuous variable (Time since 1st MSK consultation) |
| 4 | MSK contacts-2 | Continuous variable (Time since last MSK consultation) |
| 5 | Frequency of MSK contacts | Continuous variable (number of MKS consultations) |
| 6 | Any analgesics prescription | Binary variable, (having the any analgesics VS. no analgesics) |
| 7 | Highest-level of analgesics prescription | Categorical variable analgesics (Highest level of any basic analgesics/weak opioid/moderate opioid/strong opioid)  0=No analgesics prescription;  1=Basic analgesics prescription;  2=Weak analgesics prescription;  3=Moderate analgesics prescription;  4=Strong or very strong analgesics prescription  Four dummy variables were introduced in the model with `0’ as the reference group |
| 8 | NSAIDS prescription | Binary variable, (having NSAIDS VS. no NSAIDS) |
| 9 | Antidepressant prescription | Binary variable, (having antidepressant VS. no antidepressant) |
| 10 | Sedative prescription | Binary variable, (having sedative VS. no sedative) |
| 11 | Muscle relaxant prescription | Binary variable, (having muscle relaxant VS. no muscle relaxant) |
| 12 | Any MSK referral | Binary variable, (having any MSK referral vs. no MSK referral) |
| 13 | MSK X-ray | Binary variable, (having any MSK X-ray vs. no MSK X-ray) |
| 14 | MSK CT | Binary variable, (having any MSK CT vs. no MSK CT) |
| 15 | MSK MRI | Binary variable, (having any MSK MRI vs. no MSK MRI) |
| 16 | MSK surgery | Binary variable, (having any MSK surgery vs. no MSK surgery) |
| 17 | Joint injection | Binary variable, (having joint injection vs. no joint injection) |
| 18 | Fracture | Binary variable, (having fracture vs. no fracture) |
| 19 | Impact on work | Binary variable, (having any fit notes vs. no fit notes) |
| 20 | Smoking status | Categorical variable  0=Non-smoker  1=Smoking status not recorded  2=Ex-smoker  3=Current Smoker  Non-smoker as the reference group |
| 21 | Drinking status | Categorical variable  0=Non-drinker  1=Drinking status not recorded  2=Ex-drinker  3=Current drinker  Non-drinker as the reference group |
| 22 | Index of Multiple Deprivation | Categorical variable (IMD=1/2/3/4/5)  1=IMD level 1 (most deprivation)  2=IMD level 2  3=IMD level 3  4=IMD level 4  5=IMD level 5 (Least deprivation)  Most deprivation as the reference |
| 23 | Charlson Comorbidity Index | Continuous variable |
| 24 | eFI score | Continuous variable |
| 25 | Anxiety or depressant | Binary variable (having anxiety or depression consultation VS. no anxiety or depression consultation) |
| 26 | Body mass index (BMI) category | Categorical variable  0=Having normal BMI (BMI<25 kg/m^2^)  1=BMI was not recorded  2=Overweight (BMI ≥ 25 kg/m^2^ & <30 kg/m^2^)  3=Obesity (BMI≥ 30 kg/m^2^)  Normal BMI as the reference |
| 27 | MSK site-specific pain | Binary variable: having the specific pain vs. no specific pain |
