# Supplementary material for: Estimating the population health burden of musculoskeletal conditions using primary care electronic health records
Source: Rheumatology (Oxford). 2021 Feb 9;60(10):4832–43. doi: 10.1093/rheumatology/keab109 (PMC8487274; doi:10.1093/rheumatology/keab109)
Supplement: keab109_supplementary_data [file keab109_supplementary_data.zip › rhe-20-2578-File004.docx]

**Supplementary Figure-1.** Model fit statistics and model performance for multivariable prediction models based on 1-10 years’ look-back periods

Data source: PRELIM Survey-EHR data

**(a) MSK Health Indicator = High Impact Chronic Pain**


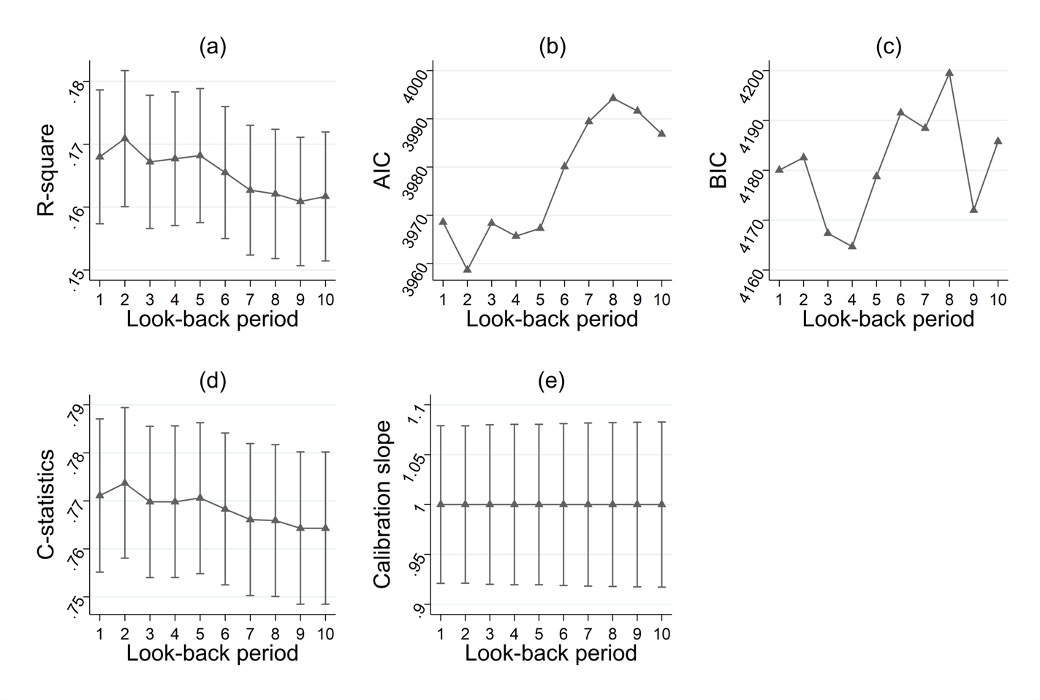


**(b) MSK Health Indicator = MSK-HQ Score**

**
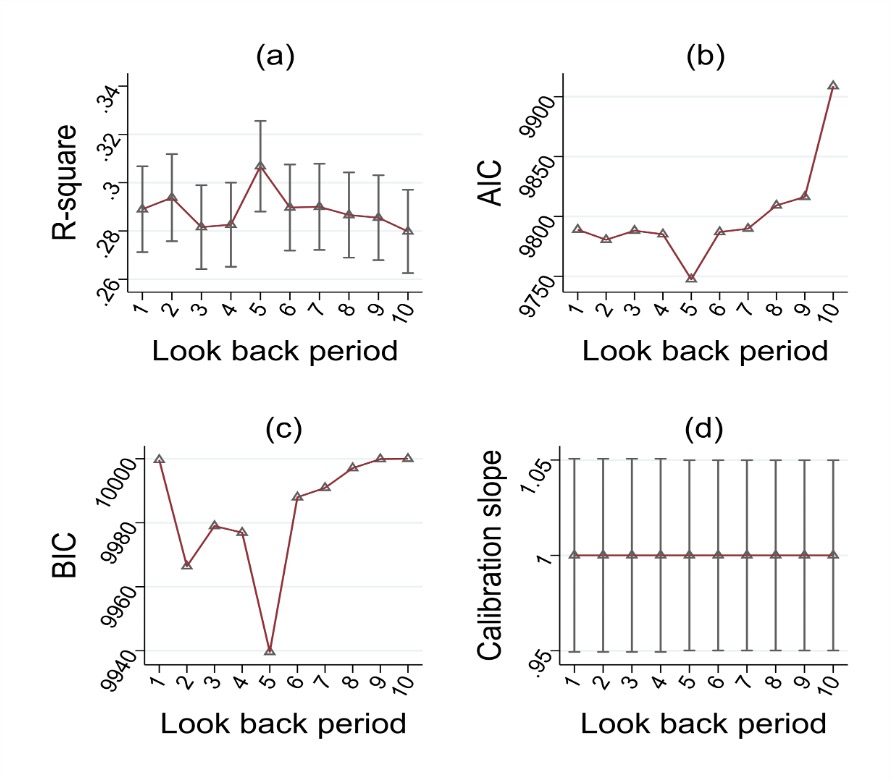
**

**(c) MSK Health Indicator = EQ-5D-5L Score**


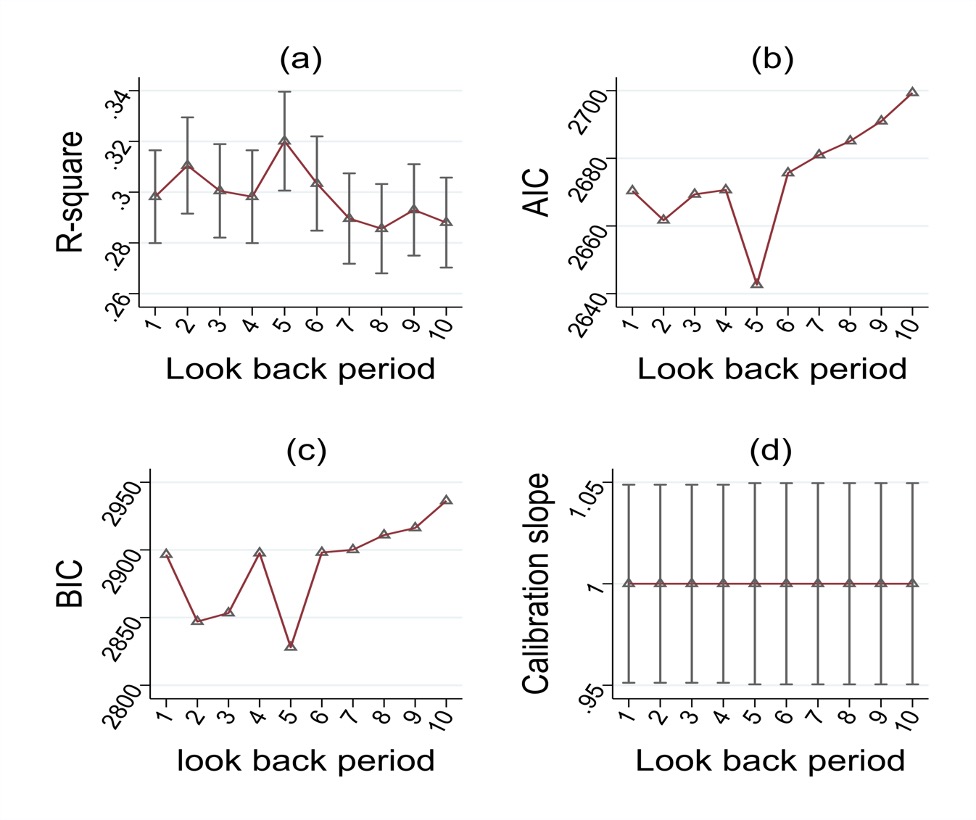


**(d) MSK Health Indicator = Moderate-to-severe chronic low back pain**

**
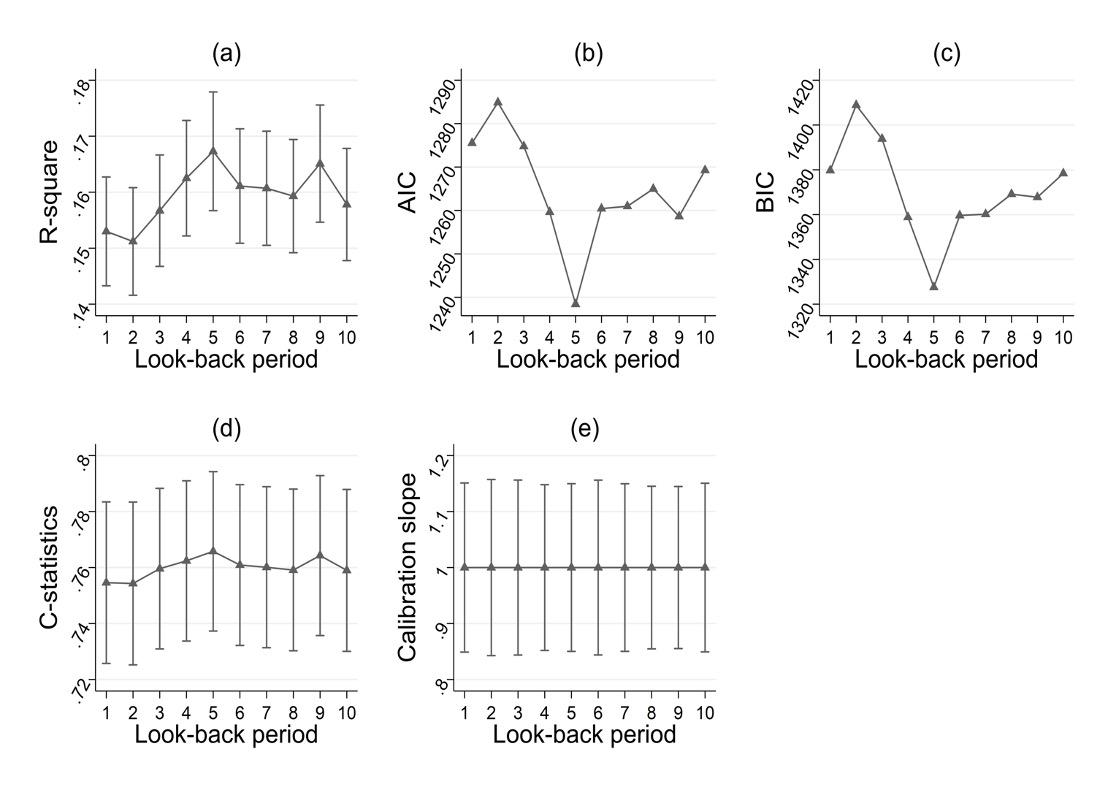
**

**(e) MSK Health Indicator = Moderate-to-severe chronic shoulder pain**

**
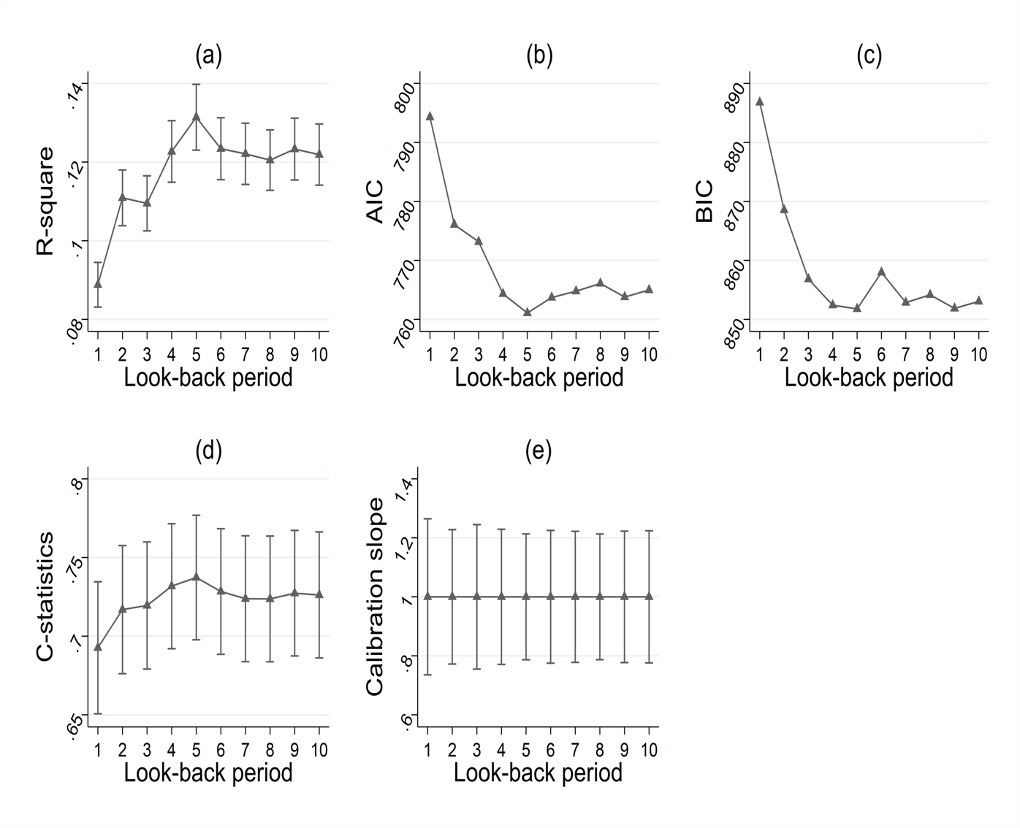
**
