# Supplementary material for: Estimating the population health burden of musculoskeletal conditions using primary care electronic health records
Source: Rheumatology (Oxford). 2021 Feb 9;60(10):4832–43. doi: 10.1093/rheumatology/keab109 (PMC8487274; doi:10.1093/rheumatology/keab109)
Supplement: keab109_supplementary_data [file keab109_supplementary_data.zip › rhe-20-2578-File005.docx]

| **Supplementary Table-2.** Final model coefficients for models based on 5-year (main analysis) and 2-year (sensitivity analysis) look-back periods   1. Models based on 5-year look-back period (main analysis)   *Polynomial term 1 for age is* ${(\frac{age}{10})}^{3}$*; polynomial term 2 for age is*$\left( \frac{age}{10} \right)^{3}\times ln(\frac{age}{10})$ *.*  *Polynomial term 1 for time since last MSK consultation is* ${(\frac{time since last MSK consultation+1}{100})}^{3}$*; polynomial term 2 for time since last MSK consultation is* $\left( \frac{time since last MSK consultation+1}{100} \right)^{3}\times ln(\frac{time since last MSK consultation+1}{100})$*.*  *Polynomial term 1 for time since first MSK consultation is* ${(\frac{time since first MSK consultation+1}{1000})}^{-2}$*; polynomial term 2 for time since first MSK consultation is* $\left( \frac{time since first MSK consultation+1}{1000} \right)^{-2}\times ln(\frac{time since first MSK consultation+1}{1000})$*.*  *Polynomial term 1 for Charlson comorbidity index is* ${(\frac{Charlson comorbidity index+1}{10})}^{-2}$*; Polynomial term 2 for Charlson comorbidity index is* ${(\frac{Charlson comorbidity index+1}{10})}^{3}$*.*  *Polynomial term 1 for eFrailty score is* ${(eFrailty score+4.028022229671478)}^{2}$*; polynomial term 2 for eFrailty score is* $\left( eFrailty score+4.028022229671478 \right)^{3}\times ln(eFrailty score+4.028022229671478)$*.*  *Polynomial term 1 for frequency of MSK consultations is* ${(\frac{frequency of MSK consultation+1}{10})}^{-2}$*; Polynomial term 2 for frequency of MSK consultations in models except moderate-to-severe back pain is* ${(\frac{frequency of MSK consultation+1}{10})}^{-0.5}$*; Polynomial term 2 for frequency of MSK consultations in model for moderate-to-severe back pain is* ${(\frac{frequency of MSK consultation+1}{10})}^{-1}$*.* | | | | | | | | | | |
| --- | --- | --- | --- | --- | --- | --- | --- | --- | --- | --- |
| **Outcomes** | **High impact chronic pain** | | **MSK HQ^^0.5^** | | **e^^EQ_5D_5L^** | | **Moderate-to-severe chronic back pain** | | **Moderate-to-severe chronic shoulder pain** | |
|  | Model coefficient | 95% CI | Model coefficient | 95% CI | Model coefficient | 95% CI | Model coefficient | 95% CI | Model coefficient | 95% CI |
| Polynomial term 1 for age | -0.010007 | (-0.020699 to 0.000686) | 0.009379 | (0.005146 to 0.013612) | 0.002243 | (0.000643 to 0.003844) | -0.014056 | (-0.032146 to 0.004034) | 0.008244 | (-0.020387 to 0.036875) |
| Polynomial term 2 for age | 0.004667 | (-0.000020 to 0.009355) | -0.004086 | (-0.005953 to -0.002220) | -0.001087 | (-0.001793 to -0.000382) | 0.006359 | (-0.001643 to 0.014361) | -0.003488 | (-0.016187 to 0.009211) |
| Gender | -0.042205 | (-0.204498 to 0.120088) | -0.012602 | (-0.077245 to 0.052041) | 0.040377 | (0.015987 to 0.064768) | -0.008924 | (-0.298181 to 0.280334) | -0.013628 | (-0.383338 to 0.356083) |
| MSK site-specific pain / condition |  |  |  |  |  |  |  |  |  |  |
| Back pain | 0.240134 | (0.065513 to 0.414755) | -0.077170 | (-0.142673 to -0.011666) | -0.013886 | (-0.038746 to 0.010974) | - | - | - | - |
| Hip pain | 0.332378 | (0.109253 to 0.5555502) | -0.171778 | (-0.248096 to -0.095460) | -0.067848 | (-0.097037 to -0.038659) | - | - | - | - |
| Knee pain | - | - | -0.160509 | (-0.227744 to -0.093274) | -0.052710 | (-0.078257 to -0.027162) | - | - | - | - |
| Osteoarthritis | 0.497058 | (0.313784 to 0.680333) | -0.143367 | (-0.218432 to -0.068303) | -0.050399 | (-0.078855 to -0.021944) | - | - | - | - |
| Polynomial term 1 for time since 1st MSK consultation, | 0.002772 | (-0.007368 to 0.012911) | -0.002521 | (-0.005803 to 0.000761) | -0.001209 | (-0.002472 to 0.000054) | - | - | 0.064885 | (-0.016375 to 0.146144) |
| Polynomial term 2 for time since 1st MSK consultation, days | 0.000401 | (-0.001067 to 0.001869) | -0.000365 | (-0.000840 to 0.000110) | -0.000175 | (-0.000358 to 0.000008) | - | - | 0.023992 | (-0.007863 to 0.055847) |
| Polynomial term 1 for time since last MSK consultation, days | -0.135877 | (-0.274625 to 0.002870) | - | - | 0.001698 | (-0.019611 to 0.023008) | 0.53019 | (0.210772 to 0.849608) | 0.311145 | (-0.090350 to 0.712641) |
| Polynomial term 2 for time since last MSK consultation, days | 0.174966 | (0.009045 to 0.340887) | - | - | -0.004724 | (-0.030272 to 0.020823) | -0.906599 | (-1.370659 to -0.442539) | -0.649025 | (-1.234058 to -0.063992) |
| Polynomial term 1 for frequency of MSK consultations, numbers | 0.035728 | (0.008107 to 0.063350) | - | - | - | - | 0.097778 | (0.055494 to 0.140061) | 0.041654 | (-0.021291 to 0.104600) |
| Polynomial term 2 for frequency of MSK consultations, numbers | -1.087254 | (-1.540366 to -0.634141) | - | - | - | - | -0.884149 | (-1.161960 to -0.606339) | -1.24942 | (-2.298504 to -0.200336) |
| Any analgesics prescription | 0.232473 | (0.009144 to 0.455801) | -0.245730 | (-0.340318 to -0.151143) | -0.079214 | (-0.115023 to -0.043404) | - | - | - | - |
| Highest-level of analgesics prescription |  | |  | |  | |  |  |  |  |
| No analgesics | Reference group | | Reference group | | Reference group | | Reference group | |  |  |
| Basic analgesics | 0.125501 | (-0.273442 to 0.524445) | 0.120891 | (-0.017097 to 0.258879) | 0.027811 | (-0.024161 to 0.079783) | 0.045795 | (-0.545682 to 0.637272) | -0.337533 | (-1.041951 to 0.366886) |
| Weak analgesics | 0.498909 | (0.113959 to 0.883859) | -0.147114 | (-0.282454 to -0.011775) | -0.056607 | (-0.107699 to -0.005514) | 0.176225 | (-0.414080 to 0.766529) | 0.165058 | (-0.558142 to 0.888259) |
| Moderate analgesics | 0.845421 | (0.468948 to 1.221893) | -0.310441 | (-0.444802 to -0.176080) | -0.109363 | (-0.160596 to -0.058129) | 0.425776 | (-0.147932 to 0.999484) | 0.572404 | (-0.136445 to 1.281252) |
| Strong / very strong analgesics | 1.134579 | (0.803507 to 1.465651) | -0.513691 | (-0.625219 to -0.402164) | -0.191204 | (-0.233169 to -0.149239) | 0.71588 | (0.246039 to 1.185721) | 0.579814 | (-0.013603 to 1.173230) |
| Antidepressant prescription | 0.516137 | (0.350558 to 0.681717) | -0.423928 | (-0.492392 to -0.355464) | -0.168719 | (-0.197655 to -0.139784) | 0.637117 | (0.345362 to 0.928871) | - | - |
| Muscle relaxant prescription | 0.963011 | (0.298628 to 1.627393) | -0.566264 | (-0.838217 to -0.294311) | -0.265911 | (-0.369361 to -0.162462) | - | - | - | - |
| Any MSK referral | - | - | - | - | - | - | - | - | 0.447432 | (0.080279 to 0.814584) |
| MSK X-ray | - | - | - | - | - | - | - | - | 0.379676 | (-0.018678 to 0.778030) |
| MSK surgery | 0.282993 | (0.063122 to 0.502864) | - | - | - | - | - | - | - | - |
| Joint injection | - | - |  | - | -0.054374 | (-0.097932 to -0.010815) | - | - | - | - |
| Smoking status |  |  |  |  |  |  | - | - | - | - |
| Non-smoker | - | - | - | - | - | - | - | - |  | Reference group |
| Smoking status not recorded | - | - | - | - | - | - | - | - | 0.194559 | (-0.542611 to 0.931728) |
| Ex-smoker | - | - |  | - | - | - | - |  | 0.643715 | (0.135930 to 1.151500) |
| Current smoker | - | - |  | - | - | - | - |  | 0.193491 | (-0.238194 to 0.625176) |
| Index of multiple deprivation (IMD) levels |  | |  | |  | |  |  |  |  |
| Most Deprived | Reference group | | Reference group | | Reference group | | Reference group | |  |  |
| level 2 | -0.485439 | (-0.750107 to -0.220771) | 0.276490 | (0.167376 to 0.385603) | 0.112600 | (0.071168 to 0.154032) | -0.754108 | (-1.241907 to -0.266308) |  |  |
| Ilevel 3 | -0.224580 | (-0.470395 to 0.021236) | 0.294030 | (0.191435 to 0.396625) | 0.116986 | (0.077961 to 0.156010) | -0.358379 | (-0.804937 to 0.088180) |  |  |
| level 4 | -0.507685 | (-0.736194 to -0.279176) | 0.364931 | (0.270737 to 0.459126) | 0.152405 | (0.116709 to 0.188101) | -0.451316 | (-0.863726 to -0.038905) |  |  |
| Least Deprived | -0.647177 | (-0.916370 to -0.377985) | 0.447526 | (0.339821 to 0.555230) | 0.184168 | (0.143534 to 0.224802) | -0.469874 | (-0.952923 to 0.013176) |  |  |
| Polynomial term 1 for Charlson Comorbidity Index | -0.000026 | (-0.000044 to 0.000008) | 0.001324 | (0.000443 to 0.002206) | - | - | - | - | -0.004044 | (-0.008879 to 0.000791) |
| Polynomial term 2 for Charlson Comorbidity Index | 0.052502 | (-0.098348 to 0.203351) | -0.567930 | (-0.974521 to -0.161338) | - | - | - | - | -0.319901 | (-2.547159 to 1.907356) |
| Polynomial term 1 for eFI score | -0.080857 | (-0.252313 to 0.090599) | 0.084825 | (0.022682 to 0.146968) | 0.040253 | (0.017064 to 0.063442) | - | - | - | - |
| Polynomial term 2 for eFI score | 0.094989 | (-0.039739 to 0.229717) | -0.094092 | (-0.145526 to -0.042657) | -0.044693 | (-0.063643 to -0.025742) | - | - | - | - |
| Anxiety or depressant consultation | - | - |  | - | -0.049455 | (-0.083390 to -0.015521) | - | - | 0.620398 | (0.156706 to 1.084090) |
| Body mass index category |  | |  | |  | |  |  |  |  |
| Normal BMI group | - | - | - | - | - | - | Reference group | |  | - |
| BMI not recorded | - | - | - | - | - | - | 0.32924 | (-0.299850 to 0.958330) | - | - |
| Overweight | - | - | - | - | - | - | 0.280714 | (-0.285789 to 0.847217) | - | - |
| Obesity | - | - | - | - | - | - | 0.662737 | (0.168535 to 1.156939) | - | - |
| Constant | -0.157354 | - | 6.064829 | - | 2.165416 | - | 0.311166 | - | -0.285655 | - |

1. *Models based on 2-year look back period (sensitivity analysis)*

*For all models:*

*Polynomial term 1 for age is* ${(\frac{age}{10})}^{3}$*; polynomial term 2 for age is*$\left( \frac{age}{10} \right)^{3}\times ln(\frac{age}{10})$ *.*

*For models (high impact chronic pain, MSK HQ^^0.5^, e^^EQ_5D_5L^, moderate-to-severe chronic shoulder pain):*

*Polynomial term 1 for time since last MSK consultation is* ${(\frac{time since last MSK consultation+1}{100})}^{3}$*; polynomial term 2 for time since last MSK consultation is* $\left( \frac{time since last MSK consultation+1}{100} \right)^{3}\times ln(\frac{time since last MSK consultation+1}{100})$*.*

*Polynomial term 1 for time since first MSK consultation is* ${(\frac{time since first MSK consultation+1}{100})}^{3}$*; polynomial term 2 for time since first MSK consultation is* $\left( \frac{time since first MSK consultation+1}{100} \right)^{3}\times ln(\frac{time since first MSK consultation+1}{100})$*.*

*Polynomial term 1 for frequency of MSK consultations is* ${(\frac{frequency of MSK consultation+1}{10})}^{-2}$*; Polynomial term 2 for frequency of MSK consultations is* ${(\frac{frequency of MSK consultation+1}{10})}^{-2}\times ln(\frac{frequency of MSK consultation+1}{10})$*.*

*Polynomial term 1 for eFrailty score is*$ln(eFrailty score+4.034752070903778)$*; polynomial term 2 for eFrailty score* ${\ln(eFrailty score+4.034752070903778)}^{2}$*.*

*Polynomial term 1 for Charlson comorbidity index is* ${(\frac{Chalson comorbidity index+1}{10})}^{-0.5}$*; polynomial term 2 for Charlson comorbidity index is* $\left( \frac{Charlosn comorbidity index+1}{10} \right)^{-0.5}\times ln(\frac{Charlson comorbidity index+1}{10})$

*For Moderate-to-severe chronic back pain:*

*Polynomial term 1 for time since last MSK consultation is* $\frac{time since last MSK consultation+1}{100}$*; : Polynomial term 2 for time since last MSK consultation is(* ${\frac{time since last MSK consultation+1}{100})}^{3}$*.*

*Polynomial term 1 for time since first MSK consultation is* $\frac{time since first MSK consultation+1}{100}$*; : Polynomial term 2 for time since last MSK consultation is (* ${\frac{time since first MSK consultation+1}{100})}^{3}$*.*

*Polynomial term 1 for frequency of MSK consultations is ln (*$\frac{frequency of MSK consulttion+1}{10})$*; polynomial term 2 for frequency of MSK consultations is* ${(\frac{frequency of MSK consultation+1}{10})}^{3}$*.*

*Polynomial term 1 for Charlson comorbidity index is* ${(\frac{Chalson comorbidity index+1}{10})}^{-2}$*; polynomial term 2 for Charlson comorbidity index is* ${(\frac{Chalson comorbidity index+1}{10})}^{3}$*.*

| **Outcomes** | **High impact chronic pain** | | **MSK HQ^^0.5^** | | **e^^EQ_5D_5L^** | | **Moderate-to-severe chronic back pain** | | **Moderate-to-severe chronic shoulder pain** | |
| --- | --- | --- | --- | --- | --- | --- | --- | --- | --- | --- |
|  | Model coefficient | 95% CI | Model coefficient | 95% CI | Model coefficient | 95% CI | Model coefficient | 95% CI | Model coefficient | 95% CI |
| Polynomial term 1 for age | -0.010650 | (-0.021345 to 0.000044) | 0.009484 | (0.005306 to 0.013662) | 0.002193 | (0.000616 to 0.003769) | -0.011912 | (-0.029894 to 0.006071) | 0.005575 | (-0.022754 to 0.033905) |
| Polynomial term 2 for age | 0.005069 | (0.000379 to 0.009760) | -0.004199 | (-0.006042 to -0.002357) | -0.001085 | (-0.001780 to -0.001381) | 0.005397 | (-0.002560 to 0.013354) | -0.002454 | (-0.015033 to 0.010126) |
| Gender | -0.031442 | (-0.194024 to 0.131140) | -0.028859 | (-0.092310 to 0.034591) | 0.030884 | (0.007007 to 0.054761) | 0.021135 | (-0.265527 to 0.307796) | -0.019850 | (-0.385950 to 0.346251) |
| MSK site-specific pain / condition |  |  |  |  |  |  | - | - | - | - |
| Back pain | 0.048637 | (-0.123886 to 0.221160) | -0.093297 | (-0.162954 to -0.023641) | -0.024894 | (-0.051169 to 0.001381) | - | - | - | - |
| Hip pain | 0.163962 | (-0.045045 to 0.372969) | -0.208700 | (-0.296187 to -0.121213) | -0.070439 | (-0.103765 to -0.037113) | - | - | - | - |
| Knee pain |  |  | -0.099067 | (-0.172572 to -0.025563) | -0.033587 | (-0.061388 to -0.005787) | - | - | - | - |
| Osteoarthritis | 0.323738 | (0.137190 to 0.510286) | -0.172924 | (-0.251725 to -0.094122) | -0.047621 | (-0.077516 to -0.017726) | - | - | - | - |
| Polynomial term 1 for time since 1st MSK consultation, | -0.018553 | (-0.029455 to -0.007652) | 0.007622 | (0.003512 to 0.011733) | 0.002978 | (0.001391 to 0.004565) | -0.332305 | (-0.578442 to -0.086167) | -0.034621 | (-0.059669 to -0.009573) |
| Polynomial term 2 for time since 1st MSK consultation, days | 0.008974 | (0.003525 to 0.014424) | -0.003985 | (-0.006062 to -0.001908) | -0.001543 | (-0.002345 to -0.000742) | 0.003544 | (-0.000163 to 0.007251) | 0.016577 | (0.004007 to 0.029148) |
| Polynomial term 1 for time since last MSK consultation, days | -0.067851 | (-0.207242 to 0.071540) | - | - | -0.001713 | (-0.022517 to 0.019091) | 1.756165 | (0.805207 to 2.707123) | 0.450404 | (0.053170 to 0.847639) |
| Polynomial term 2 for time since last MSK consultation, days | 0.103810 | (-0.063081 to 0.270701) | - | - | -0.000574 | (-0.025534 to 0.024387) | -0.41196 | (-0.602369 to -0.221551) | -0.79285 | (-1.372336 to -0.213364) |
| Polynomial term 1 for frequency of MSK consultations, numbers | -0.132135 | (-0.183310 to -0.080959) | - | - | - | - | 0.805999 | (0.455282 to 1.156716) | -0.23818 | (-0.470948 to -0.005412) |
| Polynomial term 2 for frequency of MSK consultations, numbers | -0.060333 | (-0.085833 to -0.034783) | - | - | - | - | -0.072585 | (-0.194570 to 0.049401) | -0.12169 | (-0.251942 to 0.008562) |
| Any analgesics prescription | 0.144217 | (-0.061093 to 0.349526) | -0.215446 | (-0.307290 to -0.123602) | -0.082243 | (-0.117013 to -0.047473) | - | - | - | - |
| Highest-level of analgesics prescription |  | |  | |  | |  |  |  |  |
|  |  | |  | |  | |  |  |  |  |
| No analgesics | Reference group | | Reference group | | Reference group | | Reference group | | Reference group | |
| Basic analgesics | 0.236631 | (-0.097389 to 0.570651) | 0.043926 | (-0.083418 to 0.171270) | 0.011387 | (-0.036613 to 0.059387) | 0.323488 | (-0.204720 to 0.851696) | 0.063512 | (-0.525034 to 0.652058) |
| Weak analgesics | 0.414911 | (0.091431 to 0.738391) | -0.171008 | (-0.298073 to -0.043943) | -0.055655 | (-0.103557 to -0.007752) | 0.103506 | (-0.435865 to 0.642877) | 0.535481 | (-0.116359 to 1.187321) |
| Moderate analgesics | 0.909050 | (0.601259 to 1.216842) | -0.366641 | (-0.491687 to -0.241596) | -0.137422 | (-0.184789 to -0.090055) | 0.620316 | (0.114128 to 1.126504) | 0.607245 | (-0.047590 to 1.262080) |
| Strong / very strong analgesics | 1.231211 | (0.977001 to 1.485422) | -0.588522 | (-0.688749 to -0.488296) | -0.223472 | (-0.261165 to -0.185779) | 0.909513 | (0.512736 to 1.306290) | 0.752698 | (0.240480 to 1.264916) |
| Antidepressant prescription | 0.678589 | (0.509391 to 0.847788) | -0.486842 | (-0.557271 to -0.416413) | -0.209228 | (-0.238189 to -0.180267) | 0.625651 | (0.328307 to 0.922994) | - | - |
| Muscle relaxant prescription | 0.931430 | (0.139062 to 1.723798) | -0.575712 | (-0.896323 to -0.255100) | -0.255029 | (-0.375107 to -0.134951) | - | - | - | - |
| Any MSK referral | - | - | - | - | - | - | 0.232778 | (-0.082734 to 0.548289) | - | - |
| MSK X-ray | - | - | - | - | - | - |  |  | 0.466491 | (0.088051 to 0.844930) |
| MSK surgery | 0.338019 | (0.069219 to 0.606818) | - | - | - | - | 0.14048 | (-0.411018 to 0.691977) | - | - |
| Joint injection | - | - | - | - | -0.076733 | (-0.132695 to -0.020771) |  |  | - | - |
| Smoking status |  |  |  |  |  |  |  |  |  |  |
| Non-smoker | - | - | - | - | - | - | - | - | Reference group | |
| Smoking status not recorded | - | - | - | - | - | - | - | - | 0.307909 | (-0.174386 to 0.790204) |
| Ex-smoker | - | - | - | - | - | - | - | - | 0.638746 | (0.052483 to 1.225009) |
| Current smoker | - | - | - | - | - | - | - | - | 0.087890 | (-0.396030 to 0.571809) |
| Index of multiple deprivation (IMD) levels |  | |  | |  | |  | |  | |
|  |  | |  | |  | |  |  |  |  |
| Most Deprived | Reference group | | Reference group | | Reference group | | Reference group | |  | |
| level 2 | -0.457093 | (-0.724515 to -0.189672) | 0.282984 | (0.174883 to 0.391084) | 0.117051 | (0.076131 to 0.157971) | -0.885983 | (-1.372418 to -0.399547) | - | - |
| level 3 | -0.205939 | (-0.454168 to 0.042291) | 0.287285 | (0.185507 to 0.389063) | 0.112017 | (0.073475 to 0.150559) | -0.372693 | (-0.817865 to 0.072480) | - | - |
| level 4 | -0.459858 | (-0.691691 to -0.228024) | 0.359720 | (0.266257 to 0.453184) | 0.150393 | (0.115177 to 0.185608) | -0.388152 | (-0.802835 to 0.026531) | - | - |
| Least Deprived | -0.561226 | (-0.835813 to -0.286640) | 0.433707 | (0.326482 to 0.540932) | 0.178358 | (0.138089 to 0.218628) | -0.539186 | (-1.025632 to -0.052739) | - | - |
| Polynomial term 1 for Charlson Comorbidity Index | -1.166442 | (-3.219908 to 0.887024) | 0.710661 | (-0.149481 to 1.570802) | - | - | 0.002048 | (-0.002040 to 0.006135) | 0.652826 | (-3.982543 to 5.288195) |
| Polynomial term 2 for Charlson Comorbidity Index | -0.271787 | (-0.827604 to 0.284030) | 0.155285 | (-0.076784 to 0.387355) | - | - | 0.836969 | (-1.185555 to 2.859493) | 0.260217 | (-0.994494 to 1.514929) |
| Polynomial term 1 for eFI score | 0.702897 | (0.171162 to 1.234631) | -0.590521 | (-0.810041 to -0.371000) | -0.254807 | (-0.334471 to -0.175143) | - | - | - | - |
| Polynomial term 2 for eFI score | 0.274388 | (0.049693 to 0.499084) | -0.241158 | (-0.333534 to -0.148783) | -0.102547 | (-0.136165 to -0.068929) | - | - | - | - |
| Anxiety or depressant consultation | - | - | - | - | -0.019353 | (-0.059400 to 0.020695) | - | - | 0.367954 | (-0.192567 to 0.928475) |
| Body mass index category |  | |  | |  | |  |  |  |  |
| Normal BMI group | Reference group | | - | - | - | - | Reference group | |  |  |
| BMI not recorded | 0.059748 | (-0.255028 to 0.374524) | - | - | - | - | 0.45623 | (-0.096600 to 1.009059) | - | - |
| Overweight | 0.115882 | (-0.229211 to 0.460974) | - | - | - | - | 0.260061 | (-0.346032 to 0.866154) | - | - |
| Obesity | 0.305434 | (-0.000340 to 0.611207) | - | - | - | - | 0.939655 | (0.396727 to 1.482584) | - | - |
| Constant | 0.101733 | - | 5.606389 | - | 2.397977 | - | -0.29656 | - | -0.593468 | - |
