# Supplementary material for: Estimating the population health burden of musculoskeletal conditions using primary care electronic health records
Source: Rheumatology (Oxford). 2021 Feb 9;60(10):4832–43. doi: 10.1093/rheumatology/keab109 (PMC8487274; doi:10.1093/rheumatology/keab109)

**Supplementary Figure-2.** Visual display of non-linear associations between continuous predictors and outcomes (adjusted for all other covariates in each respective final parsimonious model)

1. **MSK Health Indicator = High Impact Chronic Pain**

*Adjusted for all other covariates in the final parsimonious models*


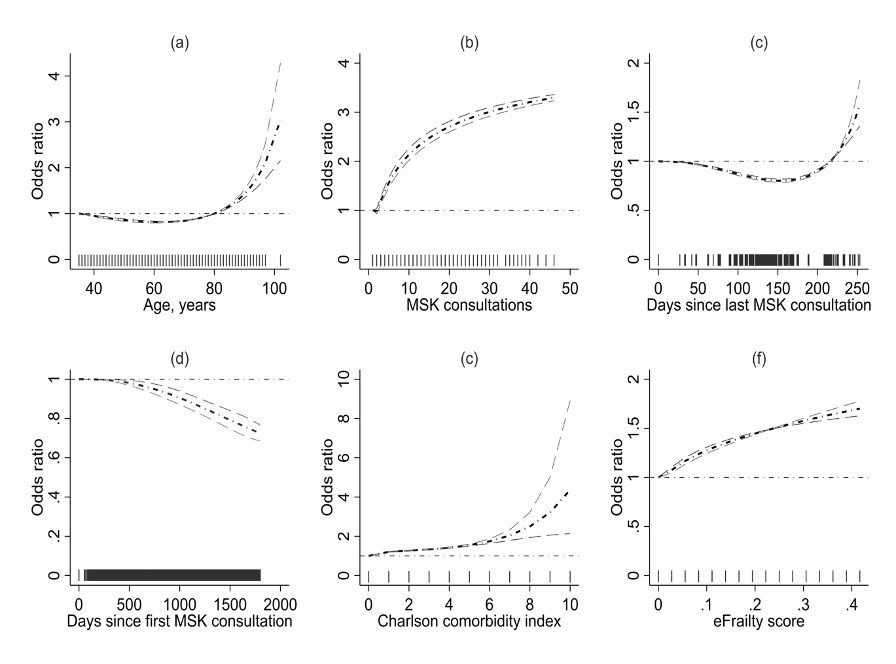


1. **MSK Health Indicator = MSK-HQ Score**

*Adjusted for all other covariates in the final parsimonious models*

**
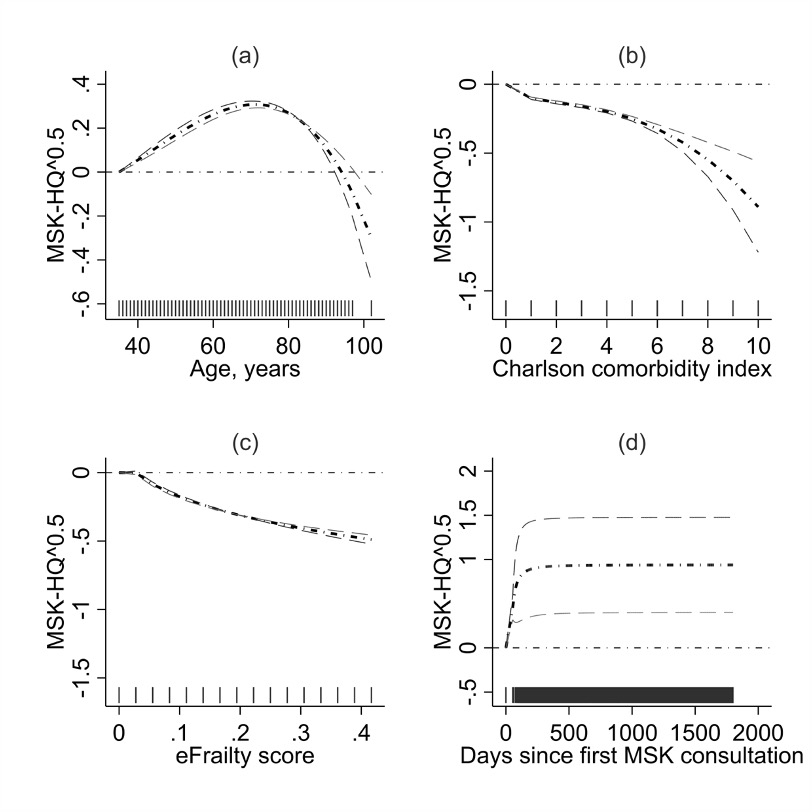
**

1. **MSK Health Indicator = EQ-5D-5L**

*Adjusted for all other covariates in the final parsimonious models*

**
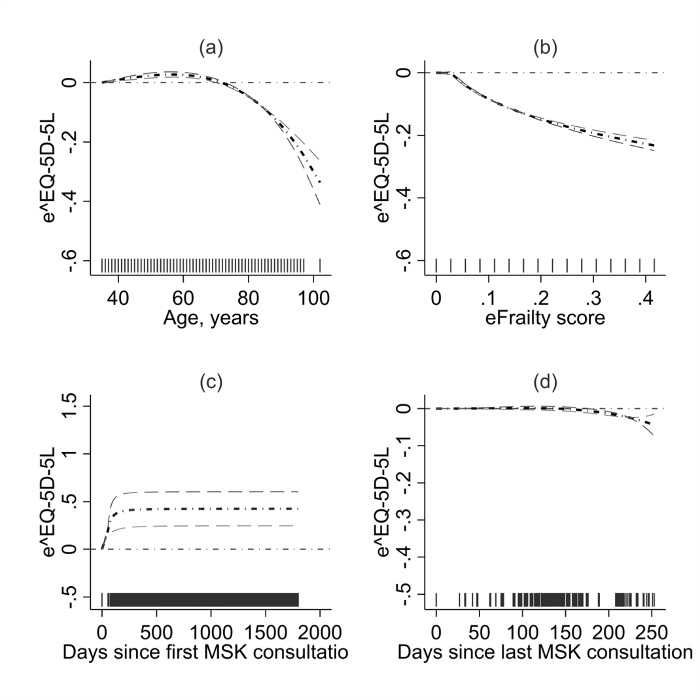
**

1. **MSK Health Indicator = Moderate-to-severe chronic low back pain**

*Adjusted for all other covariates in the final parsimonious models*

**
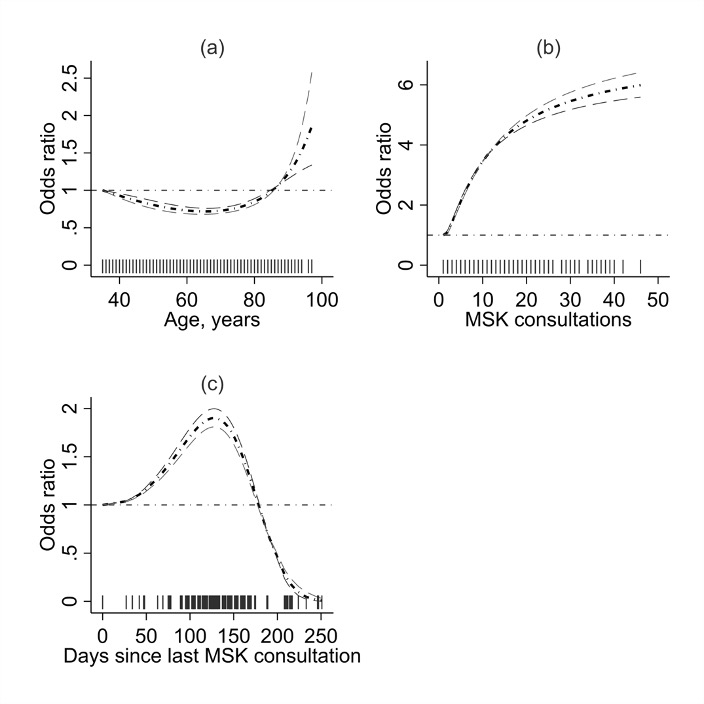
**

1. **MSK Health Indicator = Moderate-to-severe chronic shoulder pain**

*Adjusted for all other covariates in the final parsimonious models*


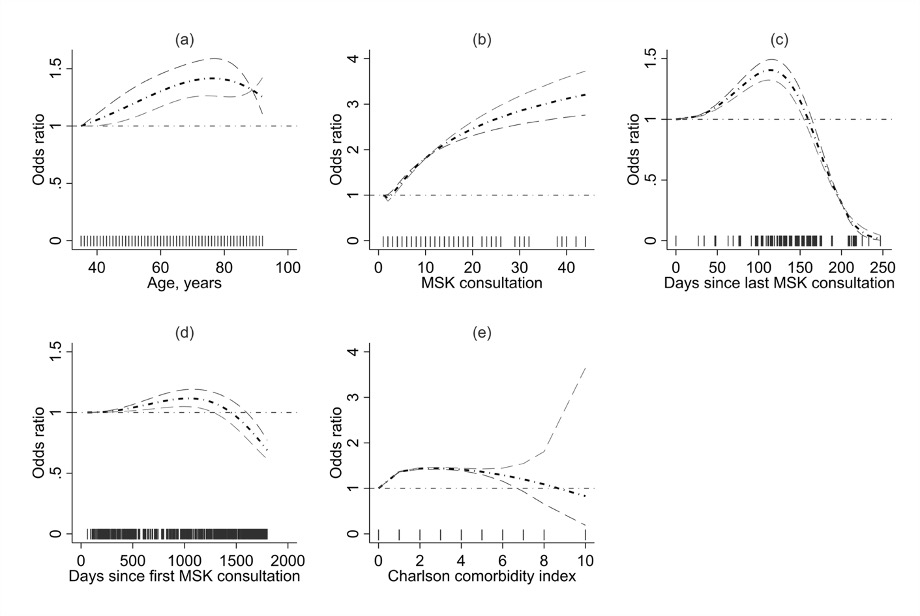

Supplement: keab109_supplementary_data [file keab109_supplementary_data.zip › rhe-20-2578-File006.docx]
