# Supplementary material for: Estimating the population health burden of musculoskeletal conditions using primary care electronic health records
Source: Rheumatology (Oxford). 2021 Feb 9;60(10):4832–43. doi: 10.1093/rheumatology/keab109 (PMC8487274; doi:10.1093/rheumatology/keab109)
Supplement: keab109_supplementary_data [file keab109_supplementary_data.zip › rhe-20-2578-File007.docx]

**Supplementary Table-3.** Model performance statistics

| **Cohort** | **MSK Health Indicator** | **Measure** | **Apparent performance** | | **Average optimism** | **Optimism-corrected** | |
| --- | --- | --- | --- | --- | --- | --- | --- |
| MSK consulters† aged 35+ years, 2016-2017 | % with high impact chronic pain | C-statistic | 0.77 | (0.75 to 0.79) | 0.0002 | 0.77 | (0.75 to 0.79) |
|  |  | Calibration slope | 1 | (0.92 to 1.08) | 0.0001 | 0.99 | (0.92 to 1.08) |
|  | Mean MSK-HQ score | R^2^ | 0.30 | (0.28 to 0.32) | 0.0016 | 0.30 | (0.28 to 0.32) |
|  |  | Calibration slope | 1 | (0.95 to 1.05) | 0.0001 | 0.99 | (0.95 to 1.05) |
|  | Mean EQ-5D-5L score | R^2^ | 0.33 | (0.31 to 0.35) | 0.0066 | 0.32 | (0.30 to 0.34) |
|  |  | Calibration slope | 1 | (0.95 to 1.05) | 0.0001 | 0.99 | (0.95 to 1.05) |
| Low back pain consulters aged 35+ years, 2016-2017 | % with moderate-to-severe chronic back pain | C-statistic | 0.77 | (0.74 to 0.79) | 0.0002 | 0.77 | (0.74 to 0.79) |
|  |  | Calibration slope | 1 | (0.92 to 1.08) | 0.0001 | 0.99 | (0.92 to 1.08) |
| Shoulder pain consulters aged 35+ years, 2016-2017 | % with moderate-to-severe chronic shoulder pain | C-statistic | 0.74 | (0.70 to 0.77) | 0.0002 | 0.74 | (0.70 to 0.77) |
|  |  | Calibration slope | 1 | (0.92 to 1.08) | 0.0001 | 0.99 | (0.92 to 1.08) |
| Data source: PRELIM Survey-EHR  † Defined as non-specific, non-inflammatory low back pain, neck pain, shoulder pain, hand/wrist pain, hip pain, knee pain, osteoarthritis  Covariates were defined using 5-year look back period (60 months prior to 30 June 2017)  See **Supplementary Table-1** for model coefficients, polynomial plots, and calibration plots for each of the 5 models.  EQ-5D-5L EuroQoL 5 dimensions, 5-level version; MSK Musculoskeletal; MSK-HQ Musculoskeletal Health Questionnaire | | | | | | | |
