# Supplementary material for: Estimating the population health burden of musculoskeletal conditions using primary care electronic health records
Source: Rheumatology (Oxford). 2021 Feb 9;60(10):4832–43. doi: 10.1093/rheumatology/keab109 (PMC8487274; doi:10.1093/rheumatology/keab109)
Supplement: keab109_supplementary_data [file keab109_supplementary_data.zip › rhe-20-2578-File008.docx]

**Supplementary Table-4**. Sensitivity analysis: England national estimates for MSK Health Indicators based on application of 2-year look period models: overall, and by age, sex, deprivation, and geographical region

| **Cohort** | MSK consulters† aged 35+ years | | | | | | | | | Low back pain consulters aged 35+ years | | | Shoulder pain consulters aged 35+ years | | |
| --- | --- | --- | --- | --- | --- | --- | --- | --- | --- | --- | --- | --- | --- | --- | --- |
| **MSK Health Indicator** | % with high impact chronic pain | | | Mean MSK-HQ score  (0-56) | | | Mean EQ-5D-5L score  (-0.224-1) | | | % with moderate-to-severe chronic back pain | | | % with moderate-to-severe chronic shoulder pain | | |
|  | 2014/5 | 2015/6 | 2016/7 | 2014/5 | 2015/6 | 2016/7 | 2014/5 | 2015/6 | 2016/7 | 2014/5 | 2015/6 | 2016/7 | 2014/5 | 2015/6 | 2016/7 |
| Overall (crude) | 30.7 | 29.7 | 28.9 | 33.8 (7.3) | 34.0 (7.4) | 34.3 (7.4) | 0.64 (0.14) | 0.64 (0.14) | 0.65 (0.15) | 30.2 | 29.1 | 29.2 | 29.8 | 27.6 | 27.7 |
|  |  |  |  |  |  |  |  |  |  |  |  |  |  |  |  |
| Men | 29.0 | 28.2 | 27.4 | 34.8 (7.2) | 35.0 (7.3) | 35.2 (7.3) | 0.64 (0.14) | 0.65 (0.14) | 0.66 (0.14) | 29.1 | 27.6 | 27.7 | 29.2 | 26.9 | 27.3 |
| Women | 31.9 | 30.7 | 30.0 | 33.0 (7.3) | 33.3 (7.4) | 33.6 (7.4) | 0.63 (0.15) | 0.64 (0.15) | 0.65 (0.15) | 31.0 | 30.1 | 30.4 | 30.3 | 28.1 | 28.0 |
|  |  |  |  |  |  |  |  |  |  |  |  |  |  |  |  |
| 35-44 years | 25.9 | 26.0 | 24.7 | 33.7 (7.3) | 33.6 (7.4) | 34.2 (7.4) | 0.67 (0.14) | 0.67 (0.14) | 0.69 (0.14) | 31.6 | 31.0 | 30.0 | 26.4 | 24.9 | 24.9 |
| 45-54 years | 25.7 | 25.5 | 24.9 | 34.7 (7.5) | 34.9 (7.6) | 35.0 (7.5) | 0.67 (0.14) | 0.68 (0.14) | 0.69 (0.14) | 29.5 | 28.6 | 29.4 | 26.9 | 25.4 | 24.9 |
| 55-64 years | 27.2 | 26.1 | 25.7 | 35.1 (7.5) | 35.3 (7.5) | 35.4 (7.6) | 0.66 (0.14) | 0.67 (0.14) | 0.68 (0.14) | 29.4 | 28.1 | 28.7 | 29.2 | 26.6 | 27.4 |
| 65-74 years | 31.2 | 30.1 | 29.6 | 34.1 (7.1) | 34.4 (7.2) | 34.5 (7.3) | 0.63 (0.13) | 0.64 (0.14) | 0.65 (0.14) | 29.5 | 28.2 | 28.1 | 31.4 | 29.2 | 29.8 |
| 75-84 years | 38.3 | 36.7 | 35.9 | 31.9 (6.6) | 32.1 (6.7) | 32.5 (6.8) | 0.58 (0.14) | 0.59 (0.14) | 0.60 (0.14) | 31.2 | 29.9 | 29.6 | 33.7 | 30.5 | 30.4 |
| 85+ years | 45.0 | 43.8 | 44.0 | 29.7 (6.0) | 29.9 (6.0) | 29.9 (6.1) | 0.53 (0.13) | 0.53 (0.13) | 0.53 (0.14) | 33.9 | 32.3 | 33.2 | 31.0 | 29.8 | 29.0 |
|  |  |  |  |  |  |  |  |  |  |  |  |  |  |  |  |
| Deprivation quintile 1 (most) | 40.7 | 40.2 | 38.8 | 28.5 (6.3) | 28.6 (6.4) | 29.0 (6.5) | 0.54 (0.14) | 0.54 (0.15) | 0.56 (0.15) | 40.3 | 39.2 | 39.7 | 33.0 | 30.2 | 29.8 |
| quintile 2 | 30.6 | 29.8 | 29.2 | 32.8 (6.8) | 32.8 (6.8) | 33.0 (6.9) | 0.62 (0.14) | 0.63 (0.14) | 0.64 (0.14) | 24.5 | 23.8 | 23.2 | 30.3 | 29.6 | 29.4 |
| quintile 3 | 33.7 | 32.7 | 32.1 | 33.5 (6.9) | 33.7 (7.0) | 33.7 (6.9) | 0.63 (0.14) | 0.63 (0.14) | 0.64 (0.14) | 30.8 | 30.5 | 30.5 | 30.2 | 28.0 | 28.4 |
| quintile 4 | 28.2 | 27.3 | 26.8 | 34.9 (7.0) | 35.0 (7.0) | 35.3 (7.1) | 0.66 (0.13) | 0.66 (0.14) | 0.67 (0.14) | 30.3 | 28.7 | 29.0 | 28.7 | 26.3 | 28.0 |
| Deprivation quintile 5 (least) | 24.9 | 24.1 | 24.0 | 36.6 (7.0) | 36.7 (7.1) | 36.9 (7.1) | 0.69 (0.13) | 0.69 (0.13) | 0.70 (0.13) | 27.1 | 26.0 | 26.1 | 28.3 | 25.9 | 25.6 |
|  |  |  |  |  |  |  |  |  |  |  |  |  |  |  |  |
| Region |  |  |  |  |  |  |  |  |  |  |  |  |  |  |  |
| North East | 35.4 | 33.9 | 36.9 | 31.8 (7.2) | 31.9 (6.9) | 31.0 (7.4) | 0.60 (0.15) | 0.61 (0.14) | 0.59 (0.16) | 34.4 | 35.7 | 38.2 | 30.3 | 30.6 | 35.4 |
| North West | 33.7 | 32.7 | 31.5 | 32.2 (7.2) | 32.4 (7.3) | 32.8 (7.2) | 0.61 (0.15) | 0.62 (0.15) | 0.63 (0.14) | 32.8 | 31.9 | 30.7 | 31.5 | 28.6 | 28.0 |
| Yorkshire & Humber | 34.3 | 28.2 | 27.1 | 32.0 (7.1) | 34.3 (7.1) | 34.5 (6.9) | 0.61 (0.15) | 0.65 (0.14) | 0.66 (0.13) | 30.6 | 26.5 | 27.6 | 30.9 | 26.4 | 25.6 |
| East Midlands | - | - | - | - | - | - | - | - | - | - | - | - | - | - | - |
| West Midlands | 31.0 | 30.8 | 30.8 | 33.7 (7.3) | 33.5 (7.3) | 33.5 (7.3) | 0.64 (0.14) | 0.63 (0.15) | 0.64 (0.15) | 31.1 | 31.1 | 31.4 | 30.4 | 28.4 | 28.4 |
| East of England | 28.5 | 26.8 | 27.4 | 34.8 (7.3) | 35.4 (7.4) | 35.1 (7.5) | 0.66 (0.14) | 0.67 (0.14) | 0.67 (0.15) | 29.2 | 27.6 | 27.1 | 29.9 | 27.3 | 27.7 |
| South West | 33.0 | 32.2 | 32.7 | 33.0 (7.4) | 33.1 (7.5) | 32.9 (7.6) | 0.62 (0.15) | 0.63 (0.15) | 0.63 (0.15) | 31.5 | 31.0 | 31.9 | 30.8 | 29.1 | 31.2 |
| South Central | 29.1 | 28.4 | 26.3 | 34.7 (7.3) | 34.9 (7.3) | 35.8 (7.2) | 0.65 (0.14) | 0.66 (0.14) | 0.68 (0.14) | 28.7 | 26.9 | 28.4 | 29.4 | 26.5 | 28.4 |
| London | 29.2 | 27.9 | 27.2 | 34.1 (7.2) | 34.3 (7.3) | 34.6 (7.3) | 0.64 (0.14) | 0.65 (0.14) | 0.66 (0.14) | 28.7 | 28.0 | 27.3 | 29.1 | 27.3 | 25.8 |
| South East Coast | 28.9 | 28.9 | 27.9 | 34.5 (7.3) | 34.5 (7.3) | 34.8 (7.4) | 0.65 (0.14) | 0.65 (0.14) | 0.66 (0.14) | 28.8 | 27.8 | 28.3 | 27.8 | 26.8 | 26.5 |
| Data source: Clinical Practice Research Datalink (CPRD)  † Defined as non-specific, non-inflammatory low back pain, neck pain, shoulder pain, hand/wrist pain, hip pain, knee pain, or osteoarthritis  Covariates were defined using 2-year look back period  EQ-5D-5L EuroQoL 5 dimensions, 5-level version; MSK Musculoskeletal; MSK-HQ Musculoskeletal Health Questionnaire | | | | | | | | | | | | | | | |
